# *Scn2a* Haploinsufficiency in Mice Suppresses Hippocampal Neuronal Excitability, Excitatory Synaptic Drive, and Long-Term Potentiation, and Spatial Learning and Memory

**DOI:** 10.3389/fnmol.2019.00145

**Published:** 2019-06-04

**Authors:** Wangyong Shin, Hanseul Kweon, Ryeonghwa Kang, Doyoun Kim, Kyungdeok Kim, Muwon Kang, Seo Yeong Kim, Sun Nam Hwang, Jin Yong Kim, Esther Yang, Hyun Kim, Eunjoon Kim

**Affiliations:** ^1^Department of Biological Sciences, Korea Advanced Institute of Science and Technology (KAIST), Daejeon, South Korea; ^2^Center for Synaptic Brain Dysfunctions, Institute for Basic Science, Daejeon, South Korea; ^3^Department of Anatomy and Division of Brain Korea 21, Biomedical Science, College of Medicine, Korea University, Seoul, South Korea

**Keywords:** sodium channel, neuronal excitability, synaptic transmission, synaptic plasticity, learning and memory, autism, intellectual disability, schizophrenia

## Abstract

Nav1.2, a voltage-gated sodium channel subunit encoded by the *Scn2a* gene, has been implicated in various brain disorders, including epilepsy, autism spectrum disorder, intellectual disability, and schizophrenia. Nav1.2 is known to regulate the generation of action potentials in the axon initial segment and their propagation along axonal pathways. Nav1.2 also regulates synaptic integration and plasticity by promoting back-propagation of action potentials to dendrites, but whether Nav1.2 deletion in mice affects neuronal excitability, synaptic transmission, synaptic plasticity, and/or disease-related animal behaviors remains largely unclear. Here, we report that mice heterozygous for the *Scn2a* gene (*Scn2a*^+/-^ mice) show decreased neuronal excitability and suppressed excitatory synaptic transmission in the presence of network activity in the hippocampus. In addition, *Scn2a*^+/-^ mice show suppressed hippocampal long-term potentiation (LTP) in association with impaired spatial learning and memory, but show largely normal locomotor activity, anxiety-like behavior, social interaction, repetitive behavior, and whole-brain excitation. These results suggest that Nav1.2 regulates hippocampal neuronal excitability, excitatory synaptic drive, LTP, and spatial learning and memory in mice.

## Introduction

Voltage-gated sodium channels play critical roles in the regulation of action potential initiation and propagation (Catterall, 2017). Mutations in the *SCN2A* gene encoding the Nav1.2 subunit of the voltage-gated sodium channel α subunit have been strongly implicated in multiple neurodevelopmental disorders (Sanders et al., 2018), including forms of epileptic disorders such as infantile epileptic encephalopathy and benign familial infantile seizures (Sugawara et al., 2001; Heron et al., 2002; Berkovic et al., 2004; Kamiya et al., 2004; Ogiwara et al., 2009; Klassen et al., 2011; Carvill et al., 2013; Epi4K Consortium et al., 2013; Nakamura et al., 2013; Touma et al., 2013; Baasch et al., 2014; Howell et al., 2015; Parrini et al., 2017; Wolff et al., 2017), autism spectrum disorders (ASD) (Weiss et al., 2003; Kamiya et al., 2004; Buxbaum et al., 2012; Sanders et al., 2012; Jiang et al., 2013; De Rubeis et al., 2014; Iossifov et al., 2014; Tavassoli et al., 2014; Codina-Sola et al., 2015; D’Gama et al., 2015; Deciphering Developmental Disorders Study, 2015; Krumm et al., 2015; Tammimies et al., 2015; Yuen et al., 2015; Turner et al., 2016; Wang T. et al., 2016; Geisheker et al., 2017; Krupp et al., 2017; Li et al., 2017; Stessman et al., 2017; Trujillano et al., 2017; Wolff et al., 2017), intellectual disability (Kamiya et al., 2004; de Ligt et al., 2012; Rauch et al., 2012; Bowling et al., 2017; Hamdan et al., 2017; Stessman et al., 2017; Wolff et al., 2017; Cherot et al., 2018; Yokoi et al., 2018), and schizophrenia (Fromer et al., 2014; Carroll et al., 2016). *SCN2A* mutations that lead to a gain of Nav1.2 function are thought to induce early onset epilepsy, whereas those that lead to a loss of Nav1.2 function induce ASD and intellectual disability (Ben-Shalom et al., 2017; Wolff et al., 2017; Sanders et al., 2018). However, the underlying pathophysiology, particularly for ASD and intellectual disability, remains largely unclear.

Nav1.2 is strongly expressed in the brain together with Nav1.1, Nav1.3, and Nav1.6 (Trimmer and Rhodes, 2004; Vacher et al., 2008; Catterall, 2017), and displays distinct spatiotemporal distribution patterns in various brain regions and at subcellular sites (Westenbroek et al., 1989; Gong et al., 1999; Boiko et al., 2001, 2003; Van Wart and Matthews, 2006; Kole et al., 2008; Hu et al., 2009; Liao et al., 2010; Li et al., 2014; Tian et al., 2014; Yamagata et al., 2017). For instance, Nav1.2 is mainly expressed in excitatory neurons in brain regions including the neocortex, hippocampus, and cerebellum (Trimmer and Rhodes, 2004; Vacher et al., 2008). Although Nav1.2 is primarily localized to axonal and nerve terminal regions, it is also detected in apical dendrites of neocortical and hippocampal pyramidal neurons (Westenbroek et al., 1989; Gong et al., 1999), as well as in the postsynaptic density of CA1 pyramidal synapses (Johnson et al., 2017).

At the neonatal stage, Nav1.2 serves as the main sodium channel subunit concentrated in the axon and axon initial segment (AIS) – a membrane specialization in proximal axons responsible for action potential generation (Bender and Trussell, 2012; Kole and Stuart, 2012). At later stages, its expression decreases in favor of Nav1.6 (Boiko et al., 2001, 2003; Kaplan et al., 2001; Liao et al., 2010; Gazina et al., 2015), which localizes to the distal side of the AIS and plays a critical role in action potential generation. At this stage, Nav1.2 comes to reside in the proximal side of the AIS and contributes to back-propagation of action potentials to dendritic and synaptic compartments (Hu et al., 2009), known to promote synaptic integration and plasticity (Magee and Johnston, 1997; Bi and Poo, 1998; Koester and Sakmann, 1998; Larkum et al., 1999; Johnston et al., 2003; Feldman, 2012; Kim et al., 2015).

Recent studies have shown that mice with a heterozygous deletion of *Scn2a* display absence-like seizure (Ogiwara et al., 2018) and impaired spatial working and reference memory, effects that are associated with altered hippocampal replay content (Middleton et al., 2018). However, although a recent study has suggested a novel role for Nav1.2 in regulating dendritic GABA release in granule cells in the olfactory bulb (Nunes and Kuner, 2018), *in vivo* evidence supporting the dendritic and synaptic roles of Nav1.2 is limited. In addition, whether mice heterozygous for *Scn2a* display behavioral phenotypes related to ASD and intellectual disability remains unclear.

In the present study, we generated a new heterozygous *Scn2a* mutant mouse line in which one allele contains a deletion of exons 4–6. We found that these mice display decreases in neuronal excitability, excitatory synaptic drive, and long-term potentiation (LTP) in the hippocampus. They also show decreased hippocampus-dependent spatial learning and memory, but largely normal locomotor activity, anxiety-like behavior, social interaction, repetitive behavior, and whole-brain excitation.

## Materials and Methods

### Animals

Floxed *Scn2a* mice in a C57BL/6J genetic background carrying a deletion of exons 4–6 of the *Scn2a* gene (encompassing the 5′ untranslated region and the first 158 amino acids of the protein) flanked by loxP sites and a neomycin cassette (*Scn2a*^cassette/+^) were designed and generated by Biocytogen. The neomycin cassette was removed by crossing *Scn2a*^cassette/+^ mice with protamine-Flp mice (C57BL/6J), yielding floxed heterozygous mice (*Scn2a*^f/+^). *Scn2a*^+/-^ mice were subsequently obtained by *in vitro* fertilization of eggs from female *Scn2a*^f/+^ mice with sperm from male C57BL/6J mice. To accelerate the generation of *Scn2a*^+/-^ mice, we treated fertilized eggs at the two-cell embryo stage with purified HTNC, a cell-permeable Cre recombinase (see below for details), in media at a final concentration of 0.3 μM for 30–40 min. After treating with HTNC, the embryos were washed and transferred to surrogate ICR female mice. *Scn2a*^+/+^, *Scn2a*^+/-^, and *Scn2a*^-/-^ mice were genotyped by polymerase chain reaction (PCR) using the following primer sets: set 1, 5′-TGG AGC GCT GAA GTT CCT ATT-3′ (forward 1) and 5′-ATG CTG TGC TAG GGG TTG GA-3′ (reverse 1); and set 2, 5′-TGT TGG CAT TCT GCA TGA CAT T-3′ (forward 2) and 5′-AGG CAG TAC CAT TCC AAT CCA-3′ (reverse 2). Young mice were weaned at approximately postnatal day 21–27 (P21–27). After weaning, a maximum of eight littermates of mixed genotype were group-housed before experiments. Animals were housed under a 12-h (13:00–01:00) dark/light cycle and were fed *ad libitum*. All animals were bred and maintained according to the Requirements of Animal Research at KAIST, and all procedures were approved by the Committees of Animal Research at KAIST (KA2016-31).

### Expression and Purification of HTNC

The pTriEx-HTNC construct encoding HTNC (histidine-TAT-NLS-Cre), a His_6_-tagged Cre recombinase rendered cell permeable by incorporation of the cell-penetrating TAT peptide (Peitz et al., 2002), was a kind gift from Dr. Klaus Rajewsky (AddGene plasmid #13763). *Escherichia coli* strain BL21 (DE3) (Enzynomics) was transformed with the HTNC construct and cultured in Luria-Bertani (LB) medium containing 50 μg/ml ampicillin to an optical density at 600 nm (OD_600_) of 0.5–0.6, at which point expression of recombinant HTNC protein was induced by addition of 0.5 mM isopropyl-β-D-thiogalactoside (IPTG). After culturing for an additional 4 h at 37°C in the presence of IPTG, cells were harvested and resuspended in a buffer consisting of 50 mM Tris-HCl (pH 8.0), 500 mM NaCl, and 30 mM imidazole, and then lysed by sonication. HTNC proteins were initially purified using a histidine affinity column (GE Healthcare). Thereafter, the HTNC buffer was changed to an imidazole-free, low-salt buffer (50 mM Tris-HCl pH 8.0, 100 mM NaCl), and HTNC proteins were further purified by cation exchange chromatography using an SP column (GE Healthcare). Purified proteins were then exchanged into phosphate-buffered saline (PBS) using a PD-10 desalting column (GE Healthcare) and concentrated using a Centricon-YM10 centrifugal concentrator (Millipore).

### Brain Homogenates and Immunoblotting

Brain homogenates from *Scn2a*^+/+^, *Scn2a*^+/-^, and *Scn2a*^-/-^ mice were prepared as described previously (Lee et al., 2015). Briefly, mouse brains (2 months for *Scn2a*^+/+^ and *Scn2a*^+/-^ mice; embryonic day 20.5 for *Scn2a*^-/-^) were homogenized in ice-cold homogenization buffer (0.32 M sucrose, 10 mM HEPES, pH 7.4, 2 mM EDTA, protease inhibitors and phosphatase inhibitors). Brain lysates were immunoblotted with Nav1.2 antibodies (Alomone, ASC-002, 1:200 or NeuroMab, K69/3, 1:500).

### Immunohistochemistry

After cardiac perfusion of adult mice (3 months) using 1% heparin and subsequent 4% paraformaldehyde (PFA), brains were stored in 4% PFA for more than 1 day. Coronal sections (40μm), prepared using a vibratome (Leica), were blocked with 5% goat serum, 0.2% TritonX-100 for 1 h and incubated with primary antibodies (1:500 for NeuN) for 24 h. After washing with PBS three times, sections were incubated with fluorophore-conjugated secondary antibodies (1:1000) in PBS with 0.2% Triton X-100 (Jackson ImmunoResearch). After washing with PBS, sections were mounted with VECTASHIELD (Vector Laboratory), and images were acquired using an LSM-780 confocal microscope (Zeiss).

### Radioisotope *in situ* Hybridization

Mouse brain sections (14 μm thick) at embryonic day (E18) and postnatal days (P0, P7, P14, P21, and P56) were prepared using a cryostat (Leica CM 1950). Hybridization probes specific for mouse *Scn2a* mRNAs were prepared using the following regions: nt 181–480 (N-term) and nt 6060–6359 (C-term) of *Scn2a* (NM_001099298.2). Antisense riboprobes were generated using ^35^S-uridine triphosphate (UTP) and the Riboprobe system (Promega).

### Fluorescence *in situ* Hybridization

Frozen mouse brain sections (14 μm thick) were cut coronally through the hippocampal formation. Sections were thaw-mounted onto Superfrost Plus Microscope Slides (Fisher Scientific 12-550-15). The sections were fixed in 4% PFA for 10 min, dehydrated in increasing concentrations of ethanol for 5 min, and finally air-dried. Tissues were then pretreated for protease digestion for 10 min at room temperature. For RNA detection, incubations with different amplifier solutions were performed in a HybEZ hybridization oven (ACDBio) at 40°C. The probes used in this study were three synthetic oligonucleotides complementary to the nucleotide (nt) sequence 2973–4072 of Mm-Scn2a-C1, nt 464–1415 of Mm-Slc17a7/Vglut1-C2, nt 1986–2998 of Mm-Slc17a6/Vglut2-C3, nt 62–3113 of Mm-Gad1-C3, nt 552–1506 of Mm-Gad2-C2, nt 2–885 of Mm-Pvalb, nt 18–407 of Mm-SST-C3, and nt 124–1280 of Mm-VIP-C3 (ACDBio, Newark, CA, United States). The labeled probes were conjugated to Atto 550 (C1), Alexa Fluor 488 (C2), and Atto 647 (C3). The sections were hybridized at 40°C with labeled probe mixtures (C1 + C2 + C3) per slide for 2 h. Then the nonspecifically hybridized probes were removed by washing the sections, three times each in 1× wash buffer at room temperature for 2 min. Amplification steps involved sequential incubations with Amplifier 1-FL for 30 min, Amplifier 2-FL for 15 min, Amplifier 3-FL for 30 min, and Amplifier 4 Alt B-FL at 40°C for 15 min. Each amplifier solutions were removed by washing three times with 1× wash buffer for 2 min at room temperature. Fluorescent images were acquired using TCS SP8 Dichroic/CS (Leica), and the ImageJ program (NIH) was used to analyze the images.

### Brain Slices for Electrophysiology

For hippocampal electrophysiology experiments, acute sagittal brain slices (300 μm thickness for whole-cell patch and 400 μm for field recordings) of *Scn2a*^+/+^ and *Scn2a*^+/-^ mice were obtained using a vibratome (Leica VT1200) after anesthetizing animals with isoflurane (Terrell). Brains were extracted and sliced in ice-cold dissection buffer containing (in mM) 212 sucrose, 25 NaHCO_3_, 5 KCl, 1.25 NaH_2_PO_4_, 0.5 CaCl_2_, 3.5 MgSO_4_, 10 D-glucose, 1.25 L-ascorbic acid, and 2 Na-pyruvate bubbled with 95% O_2_/5% CO_2_. The slices were transferred to a recovery chamber at 32°C with normal ACSF (in mM: 125 NaCl, 2.5 KCl, 1.25 NaH_2_PO_4_, 25 NaHCO_3_, 10 glucose, 2.5 CaCl_2_, and 1.3 MgCl_2_, oxygenated with 95% O_2_/5% CO_2_). After 30-min recovery at 32°C, slices were recovered for additional 30 min at 20–25°C. For the recording, a single slice was transferred to a submerged-type chamber at 27–28°C with circulating ACSF (2 ml/min) saturated with 95% O_2_ and 5% CO_2_. Stimulation and recording pipettes were pulled from thin-walled borosilicate glass capillaries (30–0065, Harvard Apparatus) with resistance 2.5–3.5 MΩ using a micropipette electrode puller (PC-10, Narishege).

### Whole-Cell Patch

Whole-cell patch-clamp recordings of hippocampal CA1 pyramidal neurons were made using a MultiClamp 700B amplifier (Molecular Devices) and Digidata 1550 (Molecular Devices). During whole-cell patch-clamp recordings, series resistance was monitored each sweep by measuring the peak amplitude of the capacitance currents in response to short hyperpolarizing step pulse (5 mV, 40 ms); only cells with a change in <20% were included in the analysis. To measure the intrinsic excitability of hippocampal CA1 cells, recording pipettes (2.5–3.5 MΩ) were filled with an internal solution containing (in mM) 137 K-gluconate, 5 KCl, 10 HEPES, 0.2 EGTA, 10 Na-phosphocreatine, 4 Mg-ATP, and 0.5 Na-GTP, with pH 7.2, 280 mOsm. To inhibit postsynaptic responses, picrotoxin (100 μM), NBQX (10 μM) and D-AP5 (50 μM) were added. After rupturing the cell, currents were clamped, and resting membrane potential (RMP) was measured. Cells with RMP larger than -60 mV were not used. After stabilizing cells, RMP was adjusted by -65 mV. Current inputs were increased from 0 to 330 pA in increments of 30 pA per sweep. Each current was injected with the time interval of 15 s. For mEPSCs in hippocampal CA1 pyramidal neurons, recording pipettes (2.5–3.5 MΩ) were filled with an internal solution containing (in mM) 100 CsMeSO_4_, 10 TEA-Cl, 8 NaCl, 10 HEPES, 5 QX-314-Cl, 2 Mg-ATP, 0.3 Na-GTP, and 10 EGTA, with pH 7.25, 295 mOsm. Whole-cell recordings of mEPSCs were obtained in neurons at a holding potential of -70 mV. TTX (1 μM) and picrotoxin (100 μM) were added to ACSF to inhibit spontaneous action potential-mediated synaptic currents and inhibitory postsynaptic currents (IPSCs), respectively. For recordings of spontaneous excitatory postsynaptic currents (sEPSCs), only picrotoxin (100 mM) were added to ACSF. For hippocampal CA1 pyramidal neuron mIPSCs, recording pipettes (2.5–3.5 MΩ) were filled with an internal solution containing (in mM) 120 CsCl, 10 TEA-Cl, 8 NaCl, 10 HEPES, 5 QX-314-Cl, 4 Mg-ATP, 0.3 Na-GTP and 10 EGTA, with pH 7.35, 280 mOsm. TTX (1 μM), NBQX (10 μM) and D-AP5 (50 μM) were added to ACSF to inhibit spontaneous action potential-mediated synaptic currents, AMPAR-mediated currents and *N*-methyl-*D*-aspartate receptor (NMDAR)-mediated currents, respectively. For the recording of spontaneous inhibitory postsynaptic currents (sIPSCs), NBQX (10 μM) and D-AP5 (50 μM) were added to ACSF. For measuring NMDAR/AMPAR ratio, CA1 pyramidal neurons were voltage clamped at -70 mV, and EPSCs were evoked at every 15 s. AMPAR-mediated EPSCs were recorded at -70 mV, and 20 consecutive responses were recorded after stable baseline. After recording AMPAR-mediated EPSCs, holding potential was changed to +40 mV to record NMDAR-mediated EPSCs. NMDA component was measured at 60 ms after the stimulation. The NMDA/AMPA ratio was determined by dividing the mean value of 20 NMDA components of EPSCs by the mean value of 20 AMPAR-mediated EPSC peak amplitudes. Data were acquired by Clampex 10.2 (Molecular Devices) and analyzed by Clampfit 10 (Molecular Devices). Drugs were purchased from Abcam (TTX), Tocris (NBQX, D-AP5), and Sigma (picrotoxin).

### Field Recording

In field recordings, fEPSPs were recorded in the stratum radiatum of the hippocampal CA1 region using pipettes filled with ACSF. fEPSPs were amplified (MultiClamp 700B, Molecular Devices) and digitized (Digidata 1550, Molecular Devices) for measurements. The Schaffer collateral pathway was stimulated, and baseline responses were collected every 20 s with a stimulation intensity that yielded a half-maximal response. For input/output experiments, after acquiring a stable baseline, a series of increasing input stimuli were given to evoke output signals. Measured fEPSP slopes and fiber volleys were then interpolated by linear fits to plot input/output relationships. For paired-pulse ratio experiments, stimuli with indicated inter-pulse intervals (25, 50, 75, 100, 200, 300 ms) were given, pairs of peak amplitudes were recorded, and the ratio of that amplitudes was calculated. To induce LTP and long-term depression (LTD) at Schaffer collateral synapses on CA1 pyramidal neurons, high-frequency stimulation (100 Hz, 1 s), theta-burst stimulation (10 trains of 4 pulses at 100 Hz), or low-frequency stimulation (1 Hz, 15 min), was applied. Data were acquired by Clampex 10.2 (Molecular Devices) and analyzed by Clampfit 10 (Molecular Devices).

### Animal Behavioral Tests

All behavioral assays were performed using littermates or age-matched male animals during light-off periods, except for automated 48-h movement analyses in LABORAS cages. All behavioral test results were performed and analyzed in a blinded manner.

### Three-Chamber Social Interaction Test

The three-chamber test (Silverman et al., 2010) was performed as described previously (Won et al., 2012; Chung et al., 2015). Briefly, a subject mouse was placed in the center region of the three-chamber apparatus, which contains a center and two side chambers. In the first session, the subject mouse was allowed to freely move around the whole three chambers for 10 min. The mouse was then gently confined in the center chamber while a novel “Object” and a wild-type (WT) stranger mouse “Stranger 1 (129Sv strain)” was placed in the containers in the two side chambers. The subject mouse was then allowed to freely explore all three chambers for 10 min. In the third session, the subject mouse was again gently guided to the center chamber while the “Object” was replaced with a WT “Stranger 2” mouse. The subject mouse was again allowed to freely explore all three chambers for 10 min.

### Direct Interaction Test and Juvenile Play Test

Each mouse was habituated in a direct social interaction box for 30 min on the day before the experiment. On test day, pairs of mice in the same age, sex, and genotype that have not met before were placed in a direct interaction box, and their interactions were recorded for 10 min. For the juvenile play test, subject mice were habituated in a new home cage with bedding for 1 h, after isolation from their mothers and siblings, on test day. Pairs of mice in the same age, sex, and genotype that have not met before were placed in a new home cage without bedding, and their interactions were recorded for 10 min. Nose-to-nose sniffing, following, mounting, and allo-grooming were quantified manually and pooled to calculate total social interaction.

### Ultrasonic Vocalization Test

An ultrasound microphone (Avisoft) and Avisoft Recorder software were used to record mice ultrasonic vocalizations (USVs). For recording adult USVs, subject male mice were placed in a home cage with an age-matched unfamiliar C57BL/6J female counterpart, and USVs were recorded for 5 min. For pup USVs, pups at the age of postnatal day 4, 6, 8, and 10 were separated from dams and placed in a glass container, and USVs were recorded for 3 min. Recorded USVs were analyzed as previously described (Kim et al., 2018). Briefly, Avisoft SASLab Pro software (**RRID:SCR_014438**) was used to analyzed USVs. Signals were filtered from 1 Hz to 100 kHz and digitized with a sampling frequency of 250 kHz, 16 bits per sample (Avisoft UltraSoundGate 116H). To generate spectrograms, the following parameters were used: FFT length, 256; frame size, 100; window, FlatTop; overlap, 75%, which resulted in a frequency resolution of 977 Hz and a temporal resolution of 0.256 ms. Frequencies lower than 45 kHz were filtered out to reduce background white noises.

### Repetitive Behaviors Test

For repetitive behaviors tests using adult mice, a subject mouse was placed in a novel and transparent grooming chamber (40 cm × 15 cm × 15 cm), and their behaviors were recorded through transparent side faces for 20 min. Self-grooming and rearing behaviors from the last 10 min were quantified manually. For juvenile repetitive behaviors, subject mice were placed in a new home cage without bedding, and their behaviors were recorded for 15 min. Self-grooming behavior from the last 10 min was quantified manually.

### Open-Field Test

Mice were placed in an open field box (40 cm × 40 cm × 40 cm) and recorded for 60 min (20 min for juvenile open-field test). The center zone line was 10 cm apart from the edge. The testing room was illuminated at ∼100 lux. Mice movements were analyzed using EthoVision XT 10 program (Noldus).

### Automated 48-h Movement Analysis (LABORAS Test)

For a long-term and real-time movement analysis, we used the LABORAS system (Metris), designed to detect and analyze vibrations delivered from a cage to a carbon-fiber vibration-sensitive plate placed underneath the cage with a mouse. Each mouse was placed in the LABORAS cage without habituation. After recording for 96 h, the data from all 96 h were analyzed using LABORAS software.

### Elevated Plus-Maze Test

The elevated plus-maze consisted of two open arms, two closed arms, and a center zone, and was elevated to a height of 50 cm above the floor. Mice were placed in the center zone and allowed to explore the space for 8 min. The data was analyzed using EthoVision XT 10 program (Noldus).

### Light–Dark Chamber Test

The apparatus for the light–dark test consisted of light (∼300 lux) and dark (∼0 lux) chambers adhered to each other. The size of the light chamber was 20 cm × 30 cm × 20 cm, and that of the dark chamber was 20 cm × 13 cm × 20 cm. An entrance enabled mice to freely move across the light and dark chambers. Mice were introduced to the center of the light chamber and allowed to explore the apparatus freely for 10 min. The time spent in dark and light chambers and the number of transitions were measured using EthoVision XT 10 program (Noldus).

### Seizure Susceptibility Test

Immediately before behavioral tests, the subject mouse received an intraperitoneal injection of pentylenetetrazol (PTZ) (40 mg/kg), or the same volume of saline, and was then allowed to acclimate to a novel cage with bedding for 10 min under low-light (60 lux) conditions. Seizure susceptibility was measured in a blinded manner based on the following behaviors: movement slowing (Phase 1), myoclonic jerk (Phase 2), clonic and generalized tonic seizure (Phase 3), and death (Phase 4). A seizure-susceptibility score was determined according to a modified Racine scale (Ferraro et al., 1999; Naydenov et al., 2014).

### Morris Water Maze Test

Mice were trained to find the hidden platform (10 cm diameter) in a white plastic tank (120 cm diameter). Mice were given three trials per day with an inter-trial interval of 30 min. The learning phase of the water maze was performed for seven consecutive days, followed by the probe test on day 8 where mice were given 1 min to find the removed platform. For reversal training (days 9–11), the location of the platform was switched to the opposite position from the previously trained position, and mice were trained to learn the new position of the platform. Target quadrant occupancy and the exact number of crossings over the former platform location during the probe test were measured using EthoVision 10 program (Noldus).

### Rotarod Test

Mice were placed on the rotating rod for 10 s, followed by the start of rod rotation. The rotating speed of rod was gradually increased from 4 to 40 rpm over 5 min. The assay was performed for two consecutive days and three times per day, while measuring the latencies of mice falling from the rod or showing 360-degree rotation on the rod twice.

### Novel Object Recognition Test

Object recognition test was performed in the open field box. On the first day, mice were allowed to explore two identical objects for 10 min. Twenty-four hours later, mice were placed the same box where one of the two objects was replaced with a new one. Exploration time for each object was measured. Object exploration was defined by the mouse’s nose being oriented toward the object and came within 2 cm of it as measured by EthoVision XT 10 program (Noldus).

### Contextual Fear Conditioning Test

All experiments were carried out in a fear conditioning system (Coulbourn Instruments). Training and testing were performed in a Plexiglas chamber with a stainless steel grid floor. On the training day, mice were placed in the fear chamber and allowed to freely move around the chamber for 2 min before they received five foot shocks (2 s, 0.8 mA, 1 min apart). To measure fear conditioning, mice were re-exposed the same chamber for 5 min without foot shock 24 h, or 7 days (in a separate cohort), after training.

### Maternal Homing Test

Maternal homing test was performed as previously described (Jung et al., 2018). Juvenile mice were separated from their mothers for at least 30 min before testing. The testing consists of a nest homing phase followed by a maternal homing phase. For the nest homing phase, bedding materials from the original home cage (Home) and fresh bedding (New) were placed in the opposite corner of open field box, previously described. Subject mice were placed in one empty corner, and their behaviors were recorded for 3 min. For the maternal homing phase, an empty container and container with the mother of the subject mice were placed in the two opposite empty corners of the box after finishing 3 min nest homing phase. Subject mice were placed in corner of bedding from home cage, and their behaviors were recorded for 5 min. Time spent with bedding and time spent sniffing containers was quantified using EthoVision XT 10 program (Noldus).

### Statistics

Statistical analyses were performed using GraphPad Prism 7. Normally distributed data were analyzed using Student’s *t*-test, whereas data that did not conform to a normal distribution were analyzed using the non-parametric Mann–Whitney test. For data that were normally distributed but exhibited a significant difference in variance in the *F*-test, Welch’s correction was used. Outliers were determined using ROUT test. All details of statistical analyses, including the sex, age and number of mice, are described in Supplementary Table 1.

## Results

### Generation of *Scn2a*^+/-^ Mice and Characterization of *Scn2a* mRNA Expression

**FIGURE 1:**
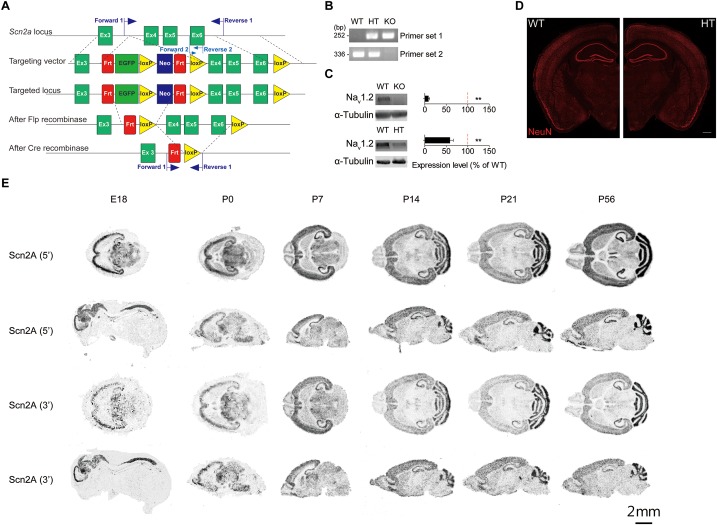
Generation of *Scn2a*^+/-^ mice and characterization of *Scn2a* mRNA expression. **(A)** Schematic diagram of the strategy for targeting exons 4–6 of the *Scn2a* gene. The two sets of primers used for PCR genotyping are indicated. **(B)** PCR genotyping of *Scn2a*^+/+^, *Scn2a*^+/-^, and *Scn2a*^-/-^ mice (2 months for WT and *Scn2a*^+/-^/HT mice; E20.5 for *Scn2a*^-/-^/KO mice). **(C)** Nav1.2 protein levels in whole-brain lysates from WT, *Scn2a*^-/-^, and *Scn2a*^+/-^ mice (2 months for WT and *Scn2a*^+/-^ mice; E20 for *Scn2a*^-/-^ mice). Data are presented as means ± SEM. *n* = 4 mice, ^∗∗^*P* < 0.01, Student’s *t*-test). **(D)** Normal gross brain morphology in *Scn2a*^+/-^ mice (3 months), as shown by staining for the neuronal marker, NeuN. Scale bar, 500 μm. **(E)** Distribution of *Scn2a* mRNA in various brain regions of WT mice at E18, P0, P7, P14, P21, and P56, revealed by isotope *in situ* hybridization. Note that the overall pattern of *Scn2a* mRNAs is similar using probes targeting 5′ and 3′ regions, although signals from the 5′ probe are stronger. Scale bar, 2 mm.

To determine whether *Scn2a* haploinsufficiency in mice leads to any changes in synaptic, neuronal or behavioral phenotypes, we generated *Scn2a*^+/-^ mice carrying a heterozygous deletion of the *Scn2a* gene (exons 4–6 covering aa 159–300 of Nav1.2; Figure 1A,B). This region also contains two exon 5 splice variants – 5N/neonatal and 5A/adult – that are known to show a neonatal-to-adult shift during postnatal brain development in mice and rats and are thought to differentially regulate neuronal excitability (Gustafson et al., 1993; Gazina et al., 2010, 2015). After removal of the neomycin cassette by crossing with a flippase-expressing mouse, the region containing exons 4–6 of the *Scn2a* gene was deleted by incubating fertilized eggs at the two-cell stage with purified, recombinant, cell-permeable HTNC (histidine-TAT-NLS-Cre) recombinase (Peitz et al., 2002) (see section “Materials and Methods” for details).

Immunoblot analyses of whole-brain lysates from 2-month-old mice using an anti-Nav1.2 antibody directed against aa 467–485 of Nav1.2 showed that Nav1.2 protein levels in the resulting *Scn2a*^+/-^ mice were approximately ∼60% of those in WT mice (Figure 1C). The gross morphology of the *Scn2a*^+/-^ mouse brain was normal, as determined by immunostaining for NeuN (neuronal marker) (Figure 1D). Homozygous *Scn2a*^-/-^ mice showed near complete elimination of Nav1.2 proteins and exhibited perinatal lethality, consistent with a previous report (Planells-Cases et al., 2000).

To determine the distribution pattern of *Scn2a* mRNA in the mouse brain at various developmental stages [embryonic day 18 (E18), P0, P7, P14, P21, and P56], we performed *in situ* hybridization experiments using horizontal and sagittal mouse brain sections and two independent radiolabeled probes targeting 5′ and 3′ regions of the *Scn2a* mRNA. These experiments revealed *Scn2a* mRNA signals in various brain regions, including the neocortex, hippocampus, striatum, thalamus, and cerebellum; similar results were obtained for 5′ and 3′ probes, although signals were stronger using the 5′ probe (Figure 1E).

### *Scn2a* Expression in Glutamatergic and GABAergic Neurons

Previous studies reported that Nav1.2 proteins are detected in glutamatergic neurons (Trimmer and Rhodes, 2004; Vacher et al., 2008) and GABAergic neurons with a caudal ganglionic eminence origin, such as vasoactive intestinal polypeptide (VIP)-positive neurons and reelin-positive/somatostatin (SST)-negative neurons in the neocortex and hippocampus (Yamagata et al., 2017). Another study, however, has reported that Nav1.2 is expressed in SST-positive, but not parvalbumin (PV)-positive, neurons in the neocortex (Li et al., 2014). To gain additional insights into the expression of *Scn2a* in glutamatergic and GABAergic neurons in mice at the mRNA level, we attempted double/triple fluorescence *in situ* hybridization for *Scn2a* and markers of glutamatergic (Vglut1/2) and GABAergic (Gad1/2) neurons using the RNA Scope method, which is known to substantially enhance signal amplification and suppress background (Wang et al., 2012).

**FIGURE 2:**
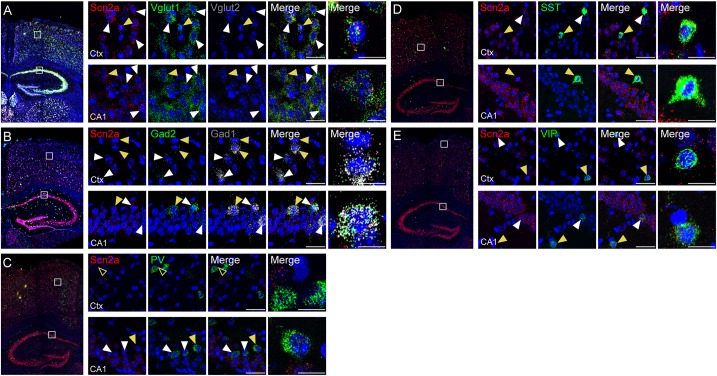
Expression of *Scn2a* mRNA in both glutamatergic and GABAergic neurons. **(A,B)** Expression of *Scn2a* mRNAs in Vglut1/2-positive glutamatergic neurons **(A)** and Gad1/2-positive GABAergic neurons **(B)** in the neocortex and hippocampus in the mouse brain (P56), as detected by fluorescence *in situ* hybridization. Coronal brain sections were triply stained for Scn2a, Vglut1/2 or Gad1/2, and DAPI (nuclear stain; blue). Images at right show enlarged views of white boxes in the images at left. Arrowheads indicate neurons that express both *Scn2a* and neuronal markers; cells indicated by yellow arrowheads were further enlarged to highlight coexpression of the markers. Scale bar, 20 and 10 μm for left and right scale bars, respectively, in each row. **(C–E)** Expression of *Scn2a* mRNA in PV-, SST-, or VIP-expressing GABAergic neurons in the neocortex and hippocampus in the mouse brain (P56), as detected by fluorescence *in situ* hybridization. Scale bar, 20 and 10 μm for left and right scale bars, respectively, in each row.

*Scn2a* mRNA was detected in both glutamatergic and GABAergic neurons in the neocortex and hippocampus of the mouse brain at P56 (Figure 2A,B). *Scn2a* mRNA was also detected in various subtypes of GABAergic neurons, including those expressing SST and VIP, although signals in PV-positive neurons were largely absent in the cortex and weak in the hippocampus (Figure 2C–E). These mRNA analysis results are partly similar to the previous reports (Li et al., 2014; Yamagata et al., 2017), although our results are from mRNA analysis. These results suggest that *Scn2a* is expressed in both glutamatergic and GABAergic neurons in the mouse brain at least at the mRNA level.

### Decreased Neuronal Excitability and Suppressed Excitatory Synaptic Transmission in the Presence of Network Activity in the *Scn2a*^+/-^ Hippocampus

Nav1.2 regulates action potential initiation and propagation in the AIS in neonatal neurons (Catterall, 2017) and back-propagation of action potentials to somatic and dendritic compartments to regulate synaptic integration and plasticity in more mature neurons (Hu et al., 2009), suggesting that *Scn2a* haploinsufficiency in mice might alter neuronal properties or synaptic functions.

**FIGURE 3:**
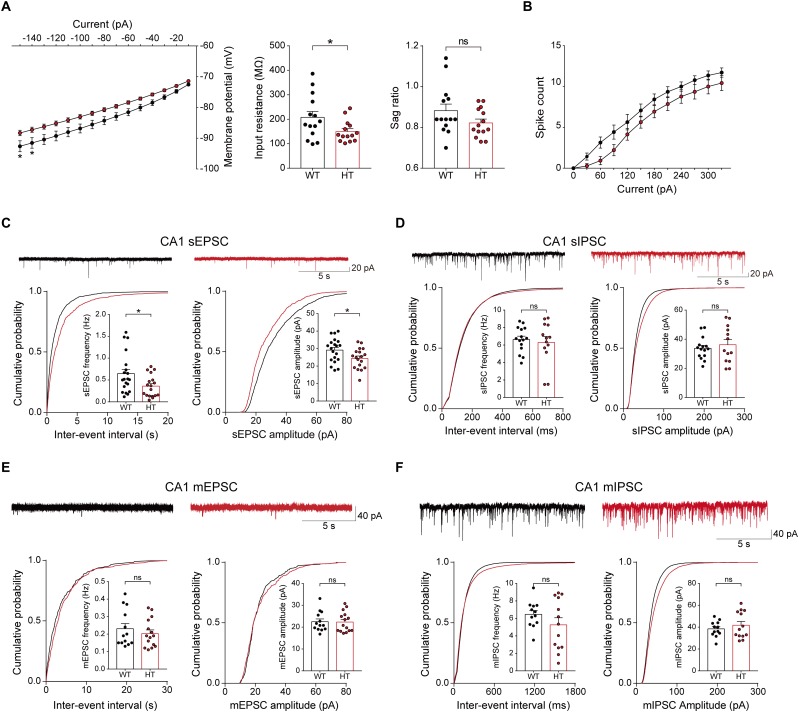
Decreased neuronal excitability and enhanced excitatory synaptic transmission in the presence of network activity in the *Scn2a*^+/-^ hippocampus. **(A,B)** Suppressed intrinsic excitability in hippocampal CA1 pyramidal neurons of *Scn2a*^+/-^ mice (3 weeks), as shown by the decrease in input resistance. Note that the sag ratio and current-spike relationship were normal, despite a decreasing trend. Data are presented as means ± SEM. *n* = 14 cells from 3 mice for WT and HT, ^∗^*P* < 0.05, ns, not significant, two-way ANOVA with Sidak’s multiple comparison test for current–voltage curve and current–firing curve, Student’s *t*-test for input resistance and sag ratio. **(C)** Suppressed sEPSC frequency and amplitude in hippocampal CA1 neurons of *Scn2a*^+/-^ mice (3 weeks). *n* = 20 cells from 4 mice for WT and 18 cells from 4 mice for HT, ^∗^*P* < 0.05, Mann–Whitney test for frequency, Student’s *t*-test for amplitude. **(D)** Normal sIPSC frequency and amplitude in hippocampal CA1 neurons of *Scn2a*^+/-^ mice (3 weeks). *n* = 15 cells from 3 mice for WT and 13 cells from 3 mice for HT, ns, not significant, Student’s *t*-test for frequency, Mann–Whitney test for amplitude. **(E)** Normal mEPSC frequency and amplitude in hippocampal CA1 neurons of Scn2a^+/-^ mice (3 weeks). *n* = 13 cells from 3 mice for WT and 15 cells from 3 mice for HT, ns, not significant, Mann–Whitney test for frequency, Student’s *t*-test for amplitude. **(F)** Normal mIPSC frequency and amplitude in hippocampal CA1 neurons of Scn2a^+/-^ mice (3 weeks). *n* = 12 cells from 3 mice for WT and 13 cells from 4 mice for HT, ns, not significant, Student’s *t*-test.

To test this, we first measured the excitability of *Scn2a*^+/-^ pyramidal neurons in the hippocampal CA1 region. *Scn2a*^+/-^ neurons showed reduced input resistance, suggesting modestly decreased intrinsic excitability, but the current-firing relationship showed only a tendency toward a decrease (Figure 3A,B).

Notably, in the presence of network activity, achieved by omitting tetrodotoxin in the recording solution, the frequency and amplitude of sEPSCs in *Scn2a*^+/-^ CA1 pyramidal neurons were significantly reduced (Figure 3C). In contrast, sIPSCs were normal in *Scn2a*^+/-^ neurons (Figure 3D). These results suggest that, in the presence of network activity, *Scn2a* haploinsufficiency suppresses intrinsic excitability and excitatory transmission, but not inhibitory synaptic transmission, in hippocampal neurons in the presence of network activity.

In contrast to these changes, miniature excitatory and inhibitory postsynaptic currents (mEPSCs and mIPSCs, respectively) were normal in *Scn2a*^+/-^ CA1 pyramidal neurons (Figure 3E,F). These results collectively suggest that excitatory network activity is strongly decreased in the hippocampus of *Scn2a*^+/-^ mice.

### *Scn2a* Haploinsufficiency Suppresses Long-Term Potentiation

Back-propagation of action potentials regulates dendritic excitability and synaptic integration and plasticity (Magee and Johnston, 1997; Bi and Poo, 1998; Koester and Sakmann, 1998; Larkum et al., 1999; Johnston et al., 2003; Feldman, 2012; Kim et al., 2015), suggesting the possibility that *Scn2a*^+/-^ SC-CA1 synapses may display altered synaptic plasticity.

**FIGURE 4:**
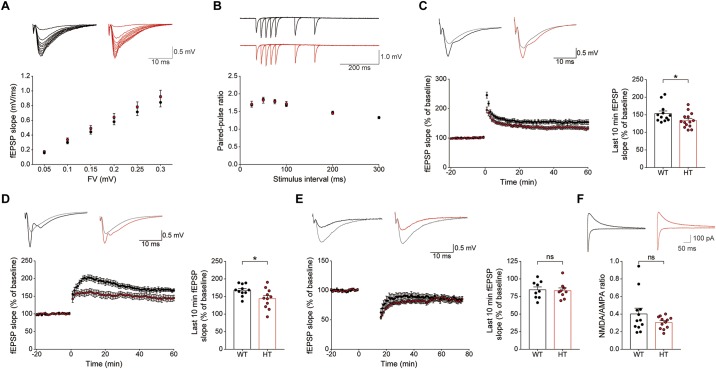
*Scn2a*^+/-^ mice display suppressed LTP, but normal LTD. **(A)** Normal basal excitatory synaptic transmission at hippocampal SC-CA1 synapses in *Scn2a*^+/-^ mice (P20–22), as shown by the input–output ratio of fEPSP slopes plotted against fiber volley (FV) amplitudes. Data are presented as means ± SEM. *n* = 10 slices from 3 mice for WT and HT, two-way ANOVA with Sidak’s multiple comparison test. **(B)** Normal paired-pulse facilitation at hippocampal SC-CA1 synapses of *Scn2a*^+/-^ mice (P20–22), as shown by fEPSP slopes plotted against inter-stimulus intervals. *n* = 10 slices from 3 mice for WT and HT, two-way ANOVA with Sidak’s multiple comparison test. **(C)** Decreased LTP induced by high-frequency stimulation (100 Hz, 1 s) at hippocampal SC-CA1 synapses of *Scn2a*^+/-^ mice (3 months). *n* = 12 slices from 6 mice for WT and 14 slices from 7 mice for HT, ^∗^*P* < 0.05, Mann–Whitney test (last 10 min). **(D)** Decreased LTP induced by theta-burst stimulation (10 trains of 4 pulses at 100 Hz) at hippocampal SC-CA1 synapses of *Scn2a*^+/-^ mice (3 months). *n* = 11 slices from 3 mice for WT and 11 slices from 4 mice for HT, ^∗^*P* < 0.05, Student’s *t*-test (last 10 min). **(E)** Normal LTD induced by low-frequency stimulation (1 Hz, 15 min) at hippocampal SC-CA1 synapses of *Scn2a*^+/-^ mice (P17–23). *n* = 9 slices from 4 mice for WT and 9 slices from 5 mice for HT, ns, not significant, Student’s *t*-test (last 10 min). **(F)** Normal NMDA/AMPA ratio at hippocampal SC-CA1 synapses of *Scn2a*^+/-^ mice (P21–25), as shown by the ratio of NMDAR- to AMPAR-mediated EPSCs. *n* = 12 cells from 5 mice for WT and 12 cells from 4 mice for HT, ns, not significant, Mann–Whitney test.

Levels of basal excitatory synaptic transmission in the Schaffer collateral-CA1 pathway (SC-CA1) were normal in the *Scn2a*^+/-^ hippocampus, as shown by the input–output relationship between fiber volley and fEPSP slope in field recordings (Figure 4A). In addition, these synapses showed normal levels of paired-pulse facilitation (Figure 4B), suggestive of unaltered presynaptic release.

An assessment of synaptic plasticity showed that LTP induced by high-frequency stimulation was suppressed at *Scn2a*^+/-^ SC-CA1 synapses (Figure 4C). Similarly, LTP-induced by theta-burst stimulation was suppressed at *Scn2a*^+/-^ SC-CA1 synapses (Figure 4D). In contrast, LTD at *Scn2a*^+/-^ SC-CA1 synapses was normal (Figure 4E).

Given that both LTP and LTD are mediated by NMDARs (Malenka and Bear, 2004; Collingridge et al., 2010), the suppressed LTP, which contrasts with the normal LTD, is unlikely to involve a decrease in NMDAR function. Indeed, in patch-clamp recordings, *Scn2a*^+/-^ SC-CA1 synapses showed a normal ratio of NMDAR- to AMPA (α-amino-3-hydroxy-5-methyl-4-isoxazolepropionic acid) receptor (AMPAR)-mediated synaptic transmission (Figure 4F). Taken together with the normal AMPAR-mediated synaptic transmission, implied by the results of spontaneous (mEPSC) and evoked (input–output) excitatory transmission (Figure 3E, 4A), this indicates that NMDAR-mediated synaptic transmission at *Scn2a*^+/-^ SC-CA1 synapses is normal. Collectively, these results suggest that *Scn2a* haploinsufficiency suppresses LTP without affecting LTD through mechanisms independent of NMDAR-mediated synaptic transmission.

### *Scn2a*^+/-^ Mice Display Impaired Spatial Learning and Memory but Enhanced Fear Memory

**FIGURE 5:**
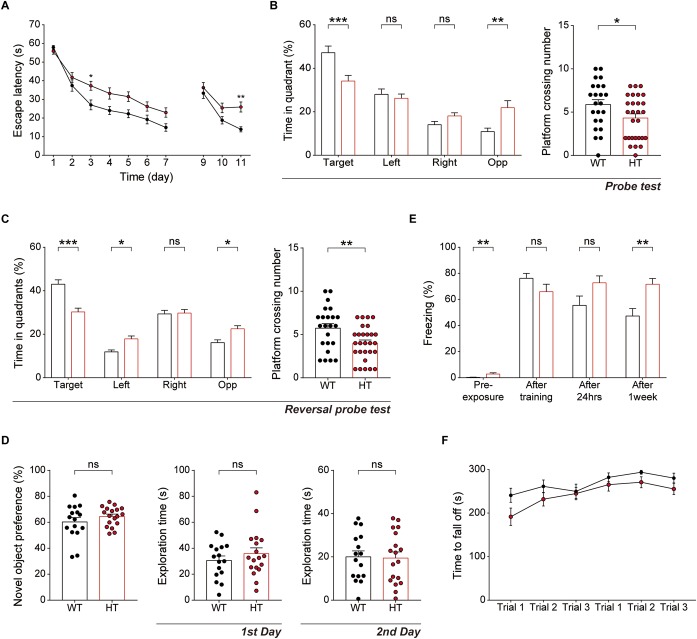
*Scn2a*^+/-^ mice show impaired spatial learning and memory and enhanced long-term fear memory. **(A–C)** Impaired spatial learning and memory in both the initial and reversal phases of the Morris water-maze test in *Scn2a*^+/-^ mice (3–4 months), as shown by escape latency, time spent in quadrant, and number of exact platform crossings in the learning phase (day 1–7), reversal phase (day 9–11), and respective probe tests (days 8 and 12). Data are presented as means ± SEM. *n* = 23 mice for WT and 28 for HT, ^∗^*P* < 0.05, ^∗∗^*P* < 0.01, ^∗∗∗^*P* < 0.001, ns, not significant, two-way ANOVA with Sidak’s multiple comparison test, Mann–Whitney test, and Student’s *t*-test. **(D)** Normal behavior of *Scn2a*^+/-^ mice (2–3 months) in novel object-recognition test, as shown by novel-object preference. Note object exploration times are normal on both first and second days. *n* = 16 mice for WT and 18 for HT, ns, not significant, Student’s *t*-test. **(E)** Normal contextual fear memory acquisition and 24-h memory in *Scn2a*^+/-^ mice (2–3 months), but enhanced 7-day fear memory in contextual fear-conditioning tests, as shown by freezing levels. Note that the 7-day experiment was performed directly after fear memory acquisition (no 24-h retrieval experiment). *n* = 13 mice for WT and 10 for HT, ^∗∗^*P* < 0.01, ns, not significant, Mann–Whitney test and Student’s *t*-test. **(F)** Normal motor learning of *Scn2a*^+/-^ mice (3–4 months) in the rotarod test, as shown by latency to fall from the rotating rod. *n* = 8 mice for WT and 12 for HT, two-way ANOVA with Sidak’s multiple comparison test.

Because hippocampal LTP is known to be associated with associative learning and memory (Bliss and Collingridge, 1993), we next subjected *Scn2a*^+/-^ mice to a battery of learning and memory tests. *Scn2a*^+/-^ mice displayed suppressed spatial learning and memory in the learning and probe phases of the Morris water-maze test compared with WT mice (Figure 5A,B). In addition, *Scn2a*^+/-^ mice performed poorly in the reversal phase of the Morris water-maze test in both learning and probe sessions (Figure 5A,C).

In the novel object-recognition test, *Scn2a*^+/-^ mice displayed novel object-recognition memory comparable to that of WT mice (Figure 5D). In the contextual fear-conditioning test, *Scn2a*^+/-^ mice showed normal memory immediately and 24 h after fear memory acquisition (Figure 5E). Intriguingly, however, these mice showed enhanced fear memory 7 days after fear memory acquisition. Therefore, these mice seem to have normal fear memory acquisition and short-term fear memory, but enhanced long-term fear memory. Lastly, *Scn2a*^+/-^ mice showed normal motor coordination and learning in the rotarod test (Figure 5F).

These results collectively suggest that *Scn2a* haploinsufficiency impairs spatial learning and memory while enhancing long-term fear memory, but does not affect object-recognition memory. In addition, *Scn2a* haploinsufficiency has mixed effects on fear memory, enhancing long-term fear memory while leaving fear-memory acquisition and short-term fear memory unaffected.

### *Scn2a*^+/-^ Mice Show Abnormally Enhanced Direct Social Interaction but Normal Social Approach, Social Communication, and Repetitive Behavior

**FIGURE 6:**
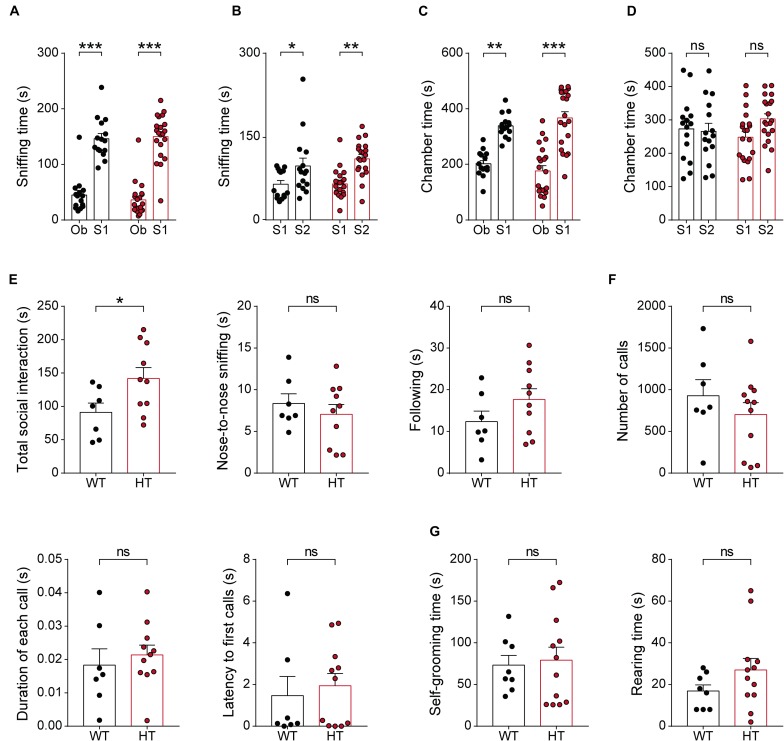
*Scn2a*^+/-^ mice show abnormally enhanced direct social interaction but normal social approach, social communication, and repetitive behavior. **(A–D)** Normal social approach in *Scn2a*^+/-^ mice (3–4 months) in the three-chamber test, as shown by time spent sniffing or in the chamber. Ob, object; S1, familiar stranger; S2, novel stranger. Data are presented as means ± SEM. *n* = 15 mice for WT and 21 for HT, ^∗^*P* < 0.05, ^∗∗^*P* < 0.01, ^∗∗∗^*P* < 0.001, ns, not significant, two-way ANOVA with Sidak’s multiple comparison test. **(E)** Enhanced social interaction in *Scn2a*^+/-^ mice (3–4 months) in the direct social-interaction test, using pairs of WT or *Scn2a*^+/-^ mice. Note that, although the total social interaction was increased, a subset of individual parameters (nose-to-nose interaction and following) was normal in *Scn2a*^+/-^ mice. *n* = 7 pairs of mice for WT and 10 for HT, ^∗^*P* < 0.05, ns, not significant, Student’s *t*-test. **(F)** Normal USVs induced by a stranger female in *Scn2a*^+/-^ mice (3–4 months), as shown by the number of USV calls, duration of each call, and latency to first call. *n* = 7 mice for WT and 11 for HT, ns, not significant, Student’s *t*-test and Mann–Whitney test. **(G)** Normal repetitive behaviors in *Scn2a*^+/-^ mice (2–3 months), as shown by time spent self-grooming and rearing. *n* = 8 mice for WT and 12 for HT, ns, not significant, Student’s *t*-test.

Given the strong association of *SCN2A* with ASD (Sanders et al., 2018), we next tested whether *Scn2a*^+/-^ mice display autistic-like impairments in social and repetitive behaviors. In the three-chamber test, known to measure social approach and social novelty-recognition behaviors in rodents (Crawley, 2004; Nadler et al., 2004; Silverman et al., 2010), *Scn2a*^+/-^ mice showed normal levels of social approach, as measured by sniffing time and time spent in the chamber (Figure 6A,C). *Scn2a*^+/-^ mice also showed normal social-novelty recognition (Figure 6B,D). In a direct social-interaction test using freely moving pairs of WT or *Scn2a*^+/-^ mice, *Scn2a*^+/-^ mice showed abnormally increased total social interaction (Figure 6E).

In tests measuring USVs, a form of social communication in rodents frequently impaired in mouse models of ASD (Scattoni et al., 2009; Wohr, 2014), *Scn2a*^+/-^ mice showed normal levels of USVs during encounters with a novel female mouse, as shown by the number of USV calls, duration of each call, and the latency to the first call (Figure 6F).

In tests measuring repetitive behaviors, *Scn2a*^+/-^ mice displayed normal levels of self-grooming and rearing (Figure 6G). These results collectively suggest that *Scn2a* haploinsufficiency induces abnormally enhanced direct social interaction, but does not affect social approach, social communication, or repetitive behavior in mice.

### *Scn2a*^+/-^ Mice Show Suppressed Locomotion in a Familiar Environment but Normal Susceptibility to Induced Seizure

Because disorders associated with *SCN2A* (epilepsy, ASD, intellectual disability, and schizophrenia) involve hyperactivity, anxiety, and seizure as important symptoms and comorbidities, we next tested locomotor behavior, anxiety-like behavior, and seizure susceptibility in *Scn2a*^+/-^ mice.

**FIGURE 7:**
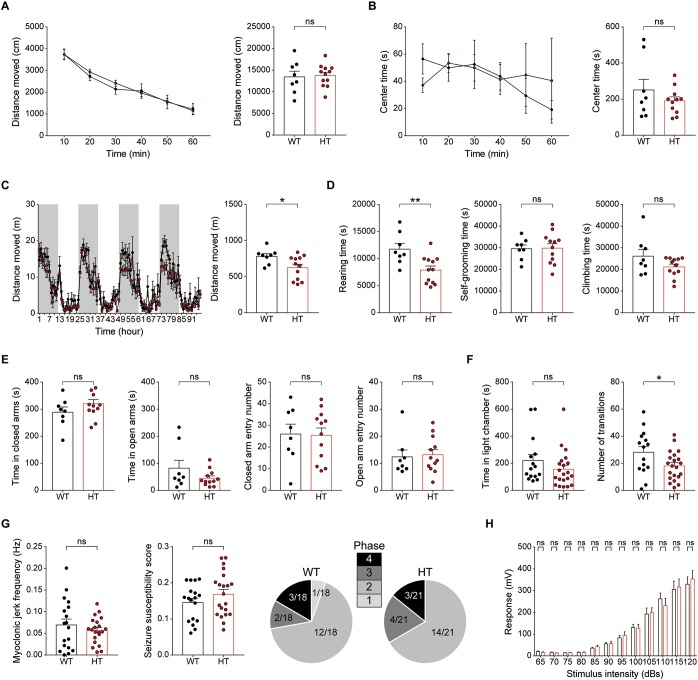
*Scn2a*^+/-^ mice show suppressed locomotion in a familiar environment, but normal anxiety-like behavior and susceptibility to induced seizure. **(A,B)** Normal locomotor activity of *Scn2a*^+/-^ mice (2–3 months) in the open-field test. Data are presented as means ± SEM. *n* = 8 mice for WT and 11 for HT, ns, not significant, Two-way ANOVA with Sidak’s multiple comparison test, Student’s *t*-test and Mann–Whitney test. **(C,D)** Decreased distance moved and rearing time, but normal self-grooming and climbing, in *Scn2a*^+/-^ mice (2–3 months) in the LABORAS test, in which mouse movements are continuously monitored for four consecutive days. Note that the extent of the decrease in distance moved became more evident as the time in LABORAS cages increased. The shaded regions indicate light-off periods. *n* = 8 mice for WT and 12 for HT, ^∗^*P* < 0.05, ^∗∗^*P* < 0.01, ns, not significant, two-way ANOVA with Sidak’s multiple comparison test, and Student’s *t*-test. **(E)** Normal anxiety-like behavior of *Scn2a*^+/-^ mice (2–3 months) in the elevated plus-maze test, as shown by time in closed/open arms and number of entries into each arm. *n* = 8 mice for WT and 12 for HT, ns, not significant, Student’s *t*-test and Mann–Whitney test. **(F)** Normal anxiety-like behavior of *Scn2a*^+/-^ mice (2–3 months) in the light–dark chamber test, as shown by time spent in the light chamber. Note that the number of transitions into the light chamber was decreased, indicative of mild hypoactivity in the light–dark apparatus. *n* = 15 mice for WT and 21 for HT, ^∗^*P* < 0.05, ns, not significant, Mann–Whitney test and Student’s *t*-test. **(G)** Normal susceptibility to PTZ-induced seizures in *Scn2a*^+/-^ mice (4 months), as shown by myoclonic jerk frequency, seizure susceptibility score, and terminal seizure stage reached. *n* = 18 mice for WT and 21 for HT, ns, not significant, Student’s *t*-test, and Chi-square test. **(H)** Normal acoustic startle response of *Scn2a*^+/-^ mice (2–3 months), as shown by the responses to different intensities of acoustic stimuli. *n* = 13 mice for WT and 10 for HT, ns, not significant, two-way ANOVA with Sidak’s multiple comparison test.

In the open-field test, *Scn2a*^+/-^ mice showed normal levels of locomotor activity and time spent in the center of the open-field arena (a measure of anxiety-like behavior) compared with WT mice (Figure 7A,B).

In LABORAS cages, in which mouse movements are measured for four consecutive days and thus represent a familiar environment (Quinn et al., 2003; Quinn et al., 2006), *Scn2a*^+/-^ mice showed decreased levels of total distance moved and time spent rearing, but normal levels of self-grooming and climbing (Figure 7C,D). Notably, the decreased locomotion became more evident as the number of days in the LABORAS cage increased, suggesting that habituation to this environment exacerbates the decreased locomotion. These results suggest that *Scn2a*^+/-^ mice display normal locomotor activity in a novel environment, but suppressed locomotor activity and repetitive behavior in a familiar environment.

*Scn2a*^+/-^ mice were then subjected to tests measuring anxiety-like behaviors. In the elevated plus-maze test, *Scn2a*^+/-^ mice showed unaltered time spent in open or closed arms and number of closed/open arm entries compared with WT mice (Figure 7E). Similarly, in the light–dark chamber test, the time spent by *Scn2a*^+/-^ mice in light/dark chambers was comparable to that of WT mice, although *Scn2a*^+/-^ mice showed mild hypoactivity in the light chamber under intense illumination (300 lux) (Figure 7F), suggestive of light-induced hypoactivity. These results suggest that *Scn2a* haploinsufficiency minimally affects anxiety-like behaviors.

Visual inspection revealed no overt evidence of seizures in *Scn2a*^+/-^ mice, an observation similar to that previously reported using an independent *Scn2a*^+/-^ mouse line with a deletion of *Scn2a* exon 1 (Planells-Cases et al., 2000; Ogiwara et al., 2018). However, because the *Scn2a*^+/-^ hippocampus showed suppressed neuronal excitability and excitatory synaptic transmission in the presence of network activity (Figure 3), we measured the susceptibility of *Scn2a*^+/-^ mice to induced seizure. *Scn2a*^+/-^ mice injected intraperitoneally with PTZ (40 mg/kg) showed a similar susceptibility to induced seizures as WT mice, as measured by myoclonic jerk frequency, seizure susceptibility score, and terminal seizure stage reached (Figure 7G). Lastly, *Scn2a*^+/-^ mice showed normal levels of acoustic startle in all sound intensity ranges tested (Figure 7H).

### Newborn and Juvenile *Scn2a*^+/-^ Mice Show Modestly Increased Direct Social Interaction and Moderately Decreased Locomotion but Normal Social Communication and Mother-Attachment Behavior

Because neurodevelopmental psychiatric disorders frequently involve early symptoms and pathophysiology, and *Scn2a* expression reaches a high level at early postnatal stages (Figure 1E), similar to results in rats (Shah et al., 2001), we subjected newborn and juvenile *Scn2a*^+/-^ mice to a set of behavioral tests.

**FIGURE 8:**
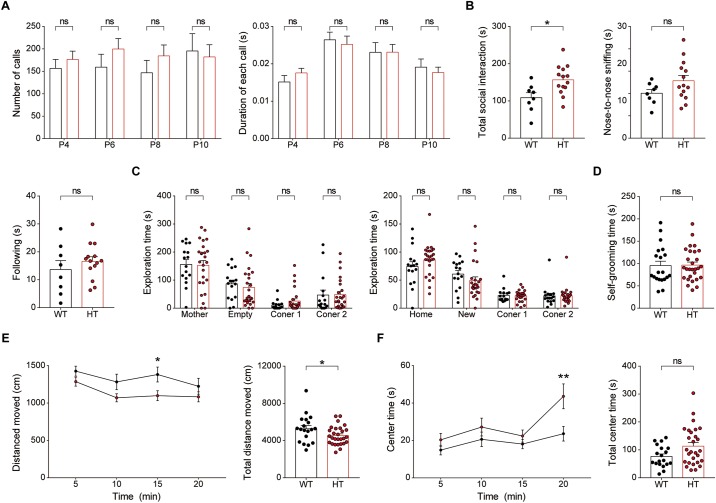
Newborn and juvenile *Scn2a*^+/-^ mice show modestly increased direct social interaction and modestly decreased locomotion but normal social communication and mother-attachment behavior. **(A)** Normal mother-seeking USVs in newborn *Scn2a*^+/-^ mice (P4–10), as shown by the number of USV calls, duration of each call, and latency to the first call. Data are presented as means ± SEM. *n* = 20 mice for WT and 27 for HT, ns, not significant, Mann–Whitney test. **(B)** Moderately increased direct social interaction (or juvenile play) in juvenile *Scn2a*^+/-^ mice (3 weeks), as indicated by nose-to-nose sniffing. Note that the total social interaction is unaltered in the mutant mice *n* = 8 mice for WT and 14 for HT, ^∗^*P* < 0.05, ns, not significant, Student’s *t*-test. **(C)** Normal mother-attachment behavior in juvenile *Scn2a*^+/-^ mice (3 weeks). *n* = 16 mice for WT and 25 for HT, ns, not significant, two-way ANOVA with Sidak’s multiple comparison test. **(D)** Normal self-grooming in juvenile *Scn2a*^+/-^ mice (3 weeks). *n* = 20 mice for WT and 28 for HT, ns, not significant, Student’s *t*-test. **(E,F)** Decreased locomotor activity and normal center time in juvenile *Scn2a*^+/-^ mice (3 weeks) in the open-field test. *n* = 19 mice for WT and 28 for HT, ^∗^*P* < 0.05, ^∗∗^*P* < 0.01, ns, not significant, two-way ANOVA with Sidak’s multiple comparison test and Student’s *t*-test.

Newborn *Scn2a*^+/-^ mice (P4–10) separated from their mother emitted USVs at levels comparable to those of WT mice, as shown by the number of USV calls and duration of each call (Figure 8A), suggesting that social communication is normal in these mice.

Juvenile *Scn2a*^+/-^ mice (∼3 weeks of age) subjected to a direct social-interaction test (also known as juvenile play) displayed moderately increased levels of direct social interaction, as shown by the significant increase in nose-to-nose sniffing (Figure 8B), similar to adult *Scn2a*^+/-^ mice, which showed increased total direct social interaction (Figure 6E). Juvenile *Scn2a*^+/-^ mice separated from their mothers for 30 min and then allowed to reunite, spent comparable amounts of time with the reunited mothers compared with WT mice (Figure 8C). Juvenile WT and *Scn2a*^+/-^ mice also showed no genotype differences in the self-grooming test (Figure 8D).

Notably, juvenile *Scn2a*^+/-^ mice showed moderately decreased locomotor activity in the open-field test (Figure 8E), a finding that contrasts with the normal open-field locomotor activity in adult *Scn2a*^+/-^ mice (Figure 7A). This suggests that the mild hypoactivity induced in juvenile *Scn2a*^+/-^ mice by a novel environment spontaneously resolves as these mice grow into adulthood. Lastly, *Scn2a*^+/-^ mice showed no anxiety-like behavior, as measured by the time spent in the center region of the open-field arena (Figure 8F).

Collectively, these results indicate that newborn and juvenile *Scn2a*^+/-^ mice show moderately increased direct social interaction and moderately decreased open-field locomotion, but normal social communication and mother-attachment behavior. In addition, the decreased open-field locomotion in young *Scn2a*^+/-^ mice contrasts with the normal open-field locomotion in adult *Scn2a*^+/-^ mice.

## Discussion

Our study demonstrates that *Scn2a* haploinsufficiency in mice leads to decreases in neuronal activity, excitatory synaptic transmission in the presence of network activity, and LTP in the hippocampus that are associated with impaired spatial learning and memory.

In support of these conclusions, our data indicate that *Scn2a*^+/-^ hippocampal CA1 neurons show moderately decreased neuronal excitability at about postnatal week 3 (Figure 3A,B). Whether this decrease is attributable to a decrease in action potential initiation or back-propagation remains unclear. Nevertheless, because Nav1.2 promotes back-propagation of action potentials, whereas Nav1.6 promotes action potential initiation in pyramidal neurons of the prefrontal cortex in P16–20 rats (Hu et al., 2009), a decrease in the back-propagation of action potentials is a possible contributor to the decreased neuronal excitability in *Scn2a*^+/-^ hippocampal neurons.

The decreased neuronal excitability in *Scn2a*^+/-^ hippocampal CA1 neurons is likely to suppress the output function of these neurons. Similar changes might also occur in neurons that lie upstream of CA1 neurons, such as CA3 and dentate gyrus neurons, as well as neocortical neurons. These changes might explain why *Scn2a*^+/-^ CA1 pyramidal neurons display a markedly decreased frequency of sEPSCs in the presence of network activity (Figure 3C,D). However, this effect does not seem to involve a decrease in excitatory synapse number because mEPSC frequency and amplitude in *Scn2a*^+/-^ CA1 pyramidal neurons was unchanged (Figure 3E).

In contrast to the normal basal excitatory synaptic transmission observed in the *Scn2a*^+/-^ hippocampus (Figure 4A,B), LTP induced by high-frequency, or theta-burst stimulation, was suppressed at *Scn2a*^+/-^ SC-CA1 synapses (Figure 4C,D). This change does not seem to involve a decrease in NMDAR function, because there was no change in NMDAR-mediated currents (Figure 4F) or LTD induced by low-frequency stimulation (Figure 4E), which, like LTP, requires NMDAR activation (Malenka and Bear, 2004; Collingridge et al., 2010). Along the same lines, the suppressed LTP is unlikely to involve post-translational modifications of NMDARs, which are known to affect NMDAR channel properties (Lussier et al., 2015). Instead, the decreased LTP likely reflects the operation of mechanisms that do not involve NMDAR-mediated synaptic currents *per se*. Importantly, back-propagation of action potentials is known to act together with dendritic sodium, calcium, and potassium channels and NMDARs to regulate the activity of dendritic properties and synaptic integration and plasticity (Magee and Johnston, 1997; Bi and Poo, 1998; Koester and Sakmann, 1998; Larkum et al., 1999; Johnston et al., 2003; Feldman, 2012; Kim et al., 2015). Therefore, the reduced Nav1.2 function in *Scn2a*^+/-^ hippocampal CA1 neurons may suppress these back-propagation processes and related synaptic plasticity.

It has been shown that distal dendrites of CA1 pyramidal neurons display local sodium spikes that are independent of back-propagating action potentials and are capable of contributing to the postsynaptic depolarization and calcium entry needed for LTP induction (Golding et al., 2002; Spruston, 2008). More recently, it has been shown that distal dendrites in freely behaving animals display local dendritic spikes and fluctuations of subthreshold membrane potentials independent of back-propagating action potentials (Moore et al., 2017). In addition, Nav1.2 signals are detectable in apical dendrites of neocortical and hippocampal pyramidal neurons (Westenbroek et al., 1989; Gong et al., 1999) as well as in the postsynaptic density of CA1 pyramidal synapses (Johnson et al., 2017). Importantly, our data indicate moderately suppressed neuronal excitability of *Scn2a*^+/-^ pyramidal neurons in the hippocampal CA1 region (Figure 3A,B). These results collectively suggest the possibility that suppressed sodium spikes and dendritic hyperpolarization in *Scn2a*^+/-^ dendrites might contribute to the suppressed LTP independent of back-propagating action potentials.

The decreased LTP in the *Scn2a*^+/-^ hippocampus is in line with the suppressed spatial learning and memory of *Scn2a*^+/-^ mice in the Morris water-maze test (Figure 5A–C). This result is also in agreement with the recently reported impairments in tasks requiring spatial working and reference memory in an independent *Scn2a*^+/-^ mouse line (deletion of exon 1 vs. deletion of exons 4–6 in our mice) (Planells-Cases et al., 2000), which are associated with altered hippocampal replay content (Middleton et al., 2018). In contrast, novel object-recognition memory was unaltered in our *Scn2a*^+/-^ mice (Figure 5D), although it should be noted that brain structures in addition to the hippocampus, such as the perirhinal cortex, have been suggested to be involved (Warburton and Brown, 2015).

Our *Scn2a*^+/-^ mice also show normal contextual-fear learning and 24-h memory (Figure 5E); these findings are seemingly at odds with Morris water-maze result, possibly reflecting differences in neural pathways or stimulus contexts/intensities between the two assays. Notably, however, *Scn2a*^+/-^ mice show abnormally enhanced 7-day fear memory (Figure 5E), suggesting that these mice are more vulnerable to strong noxious stimuli, a vulnerability that might stem from enhanced fear memory or suppressed fear memory extinction.

Behaviors associated with an autistic-like phenotype, including social interaction/communication and repetitive behaviors, were largely normal in *Scn2a*^+/-^ mice (Figure 6), a surprising result considering the strong association of *SCN2A* with ASD. It is possible that *Scn2a* haploinsufficiency in mice does not elicit autistic-like behaviors because of fundamental differences between human and mouse brains, or because the behavioral assays used are not sensitive enough to detect subtle changes in social interaction or repetitive behavior. Notably, however, *Scn2a*^+/-^ adult and juvenile mice showed increased direct social interaction (Figure 6E), a result often observed in other mouse models of autism that lack, i.e., the excitatory postsynaptic scaffolding protein Shank3 (Wang X. et al., 2016; Yoo et al., 2018). One of these studies on *Shank3*-mutant mice carrying a deletion of exons 4–22 reported that the increased social-interaction phenotype involves normal social interest but unsuccessful repetitive attempts for social interaction toward a mouse under a different genetic background (Wang X. et al., 2016), although our study used pairs of *Scn2a*^+/-^ mice in the same genetic background, making a similar analysis of unidirectional social interaction not feasible.

Lastly, susceptibility to induced seizure and acoustic startle responses were unaltered in *Scn2a*^+/-^ mice (Figure 7G,H), a finding that contrasts with the decreased neuronal excitability and excitatory synaptic drive observed in the *Scn2a*^+/-^ hippocampus. This result is similar to that obtained in a previous study using a different *Scn2a*^+/-^ mouse line (exon 1 deleted), in which seizure behaviors could not be detected by visual inspection (Planells-Cases et al., 2000). On the other hand, a recent study on this latter *Scn2a*^+/-^ mouse line employing long-term electrocorticography-electromyography recordings reported the presence of absence-like seizures with short bursts of spike-wave discharges and behavioral arrests (Ogiwara et al., 2018). This study further showed that conditional, heterozygous *Scn2a*^+/-^ mice in which deletion of yet another *Scn2a* exon (exon 2) restricted to excitatory neurons leads to similar absence-like seizures. Thus, the prediction is that electrocorticography-electromyography recordings in our mouse line might also reveal absence-like seizures, although deletion of different exons in the *Scn2a* gene might lead to different seizure phenotypes.

In conclusion, our study demonstrates that *Scn2a* haploinsufficiency in mice leads to decreases in neuronal excitability, excitatory drive, and LTP in the hippocampus that are associated with suppressed spatial learning and memory.

## Data Availability

The raw data supporting the conclusions of this manuscript will be made available by the authors, without undue reservation, to any qualified researcher.

## Ethics Statement

All animals were bred and maintained according to the Requirements of Animal Research at KAIST, and all procedures were approved by the Committees of Animal Research at KAIST (KA2016-31).

## Author Contributions

WS, HSK, RK, KK, and MK performed the behavioral experiments. WS and RK performed the immunoblot experiments. WS, HSK, and RK performed the electro-physiological experiments. DK, SK, and SH performed the HTNC experiments. JK and EY performed *in situ* hybridization experiments. WS, HK, and EK designed the experiments and wrote the manuscript.

## Conflict of Interest Statement

The authors declare that the research was conducted in the absence of any commercial or financial relationships that could be construed as a potential conflict of interest.

## References

[B1] Baasch A. L. Huning I. Gilissen C. Klepper J. Veltman J. A. Gillessen-Kaesbach G. (2014). Exome sequencing identifies a de novo SCN2A mutation in a patient with intractable seizures, severe intellectual disability, optic atrophy, muscular hypotonia, and brain abnormalities. *Epilepsia* 55 e25–e29. 10.1111/epi.12554 24579881

[B2] Bender K. J. Trussell L. O. (2012). The physiology of the axon initial segment. *Annu. Rev. Neurosci.* 35 249–265. 10.1146/annurev-neuro-062111-150339 22443507

[B3] Ben-Shalom R. Keeshen C. M. Berrios K. N. An J. Y. Sanders S. J. Bender K. J. (2017). Opposing effects on NaV1.2 function underlie differences between SCN2A variants observed in individuals with autism spectrum disorder or infantile seizures. *Biol. Psychiatry* 82 224–232. 10.1016/j.biopsych.2017.01.009 28256214 PMC5796785

[B4] Berkovic S. F. Heron S. E. Giordano L. Marini C. Guerrini R. Kaplan R. E. (2004). Benign familial neonatal-infantile seizures: characterization of a new sodium channelopathy. *Ann. Neurol.* 55 550–557. 10.1002/ana.20029 15048894

[B5] Bi G. Q. Poo M. M. (1998). Synaptic modifications in cultured hippocampal neurons: dependence on spike timing, synaptic strength, and postsynaptic cell type. *J. Neurosci.* 18 10464–10472. 10.1523/jneurosci.18-24-10464.1998 9852584 PMC6793365

[B6] Bliss T. V. Collingridge G. L. (1993). A synaptic model of memory: long-term potentiation in the hippocampus. *Nature* 361 31–39. 10.1038/361031a0 8421494

[B7] Boiko T. Rasband M. N. Levinson S. R. Caldwell J. H. Mandel G. Trimmer J. S. (2001). Compact myelin dictates the differential targeting of two sodium channel isoforms in the same axon. *Neuron* 30 91–104. 10.1016/s0896-6273(01)00265-3 11343647

[B8] Boiko T. Van Wart A. Caldwell J. H. Levinson S. R. Trimmer J. S. Matthews G. (2003). Functional specialization of the axon initial segment by isoform-specific sodium channel targeting. *J. Neurosci.* 23 2306–2313. 10.1523/jneurosci.23-06-02306.2003 12657689 PMC6742039

[B9] Bowling K. M. Thompson M. L. Amaral M. D. Finnila C. R. Hiatt S. M. Engel K. L. (2017). Genomic diagnosis for children with intellectual disability and/or developmental delay. *Genome Med.* 9:43. 10.1186/s13073-017-0433-1 28554332 PMC5448144

[B10] Buxbaum J. D. Daly M. J. Devlin B. Lehner T. Roeder K. State M. W. (2012). The autism sequencing consortium: large-scale, high-throughput sequencing in autism spectrum disorders. *Neuron* 76 1052–1056. 10.1016/j.neuron.2012.12.008 23259942 PMC3863639

[B11] Carroll L. S. Woolf R. Ibrahim Y. Williams H. J. Dwyer S. Walters J. (2016). Mutation screening of SCN2A in schizophrenia and identification of a novel loss-of-function mutation. *Psychiatr. Genet.* 26 60–65. 10.1097/YPG.0000000000000110 26555645 PMC4756433

[B12] Carvill G. L. Heavin S. B. Yendle S. C. McMahon J. M. O’Roak B. J. Cook J. (2013). Targeted resequencing in epileptic encephalopathies identifies de novo mutations in CHD2 and SYNGAP1. *Nat. Genet.* 45 825–830. 10.1038/ng.2646 23708187 PMC3704157

[B13] Catterall W. A. (2017). Forty Years of sodium channels: structure, function, pharmacology, and epilepsy. *Neurochem. Res.* 42 2495–2504. 10.1007/s11064-017-2314-9 28589518 PMC5693772

[B14] Cherot E. Keren B. Dubourg C. Carré W. Fradin M. Lavillaureix A. (2018). Using medical exome sequencing to identify the causes of neurodevelopmental disorders: Experience of 2 clinical units and 216 patients. *Clin. Genet.* 93 567–576. 10.1111/cge.13102 28708303

[B15] Chung W. Choi S. Y. Lee E. Park H. Kang J. Park H. (2015). Social deficits in IRSp53 mutant mice improved by NMDAR and mGluR5 suppression. *Nat. Neurosci.* 18 435–443. 10.1038/nn.3927 25622145

[B16] Codina-Sola M. Rodriguez-Santiago B. Homs A. Santoyo J. Rigau M. Aznar-Lain G. (2015). Integrated analysis of whole-exome sequencing and transcriptome profiling in males with autism spectrum disorders. *Mol. Autism* 6:21. 10.1186/s13229-015-0017-0 25969726 PMC4427998

[B17] Collingridge G. L. Peineau S. Howland J. G. Wang Y. T. (2010). Long-term depression in the CNS. *Nat. Rev. Neurosci.* 11 459–473. 20559335 10.1038/nrn2867

[B18] Crawley J. N. (2004). Designing mouse behavioral tasks relevant to autistic-like behaviors. *Ment. Retard. Dev. Disabil. Res. Rev.* 10 248–258. 10.1002/mrdd.20039 15666335

[B19] de Ligt J. Willemsen M. H. van Bon B. W. Kleefstra T. Yntema H. G. Kroes T. (2012). Diagnostic exome sequencing in persons with severe intellectual disability. *N. Engl. J. Med.* 367 1921–1929. 10.1056/NEJMoa1206524 23033978

[B20] De Rubeis S. He X. Goldberg A. P. Poultney C. S. Samocha K. Cicek A. E. (2014). Synaptic, transcriptional and chromatin genes disrupted in autism. *Nature* 515 209–215. 10.1038/nature13772 25363760 PMC4402723

[B21] Deciphering Developmental Disorders Study (2015). Large-scale discovery of novel genetic causes of developmental disorders. *Nature* 519 223–228. 10.1038/nature14135 25533962 PMC5955210

[B22] D’Gama A. M. Pochareddy S. Li M. Jamuar S. S. Reiff R. E. Lam A. N. (2015). Targeted DNA sequencing from autism spectrum disorder brains implicates multiple genetic mechanisms. *Neuron* 88 910–917. 10.1016/j.neuron.2015.11.009 26637798 PMC4672379

[B23] Epi4K Consortium Epilepsy Phenome/Genome Project Allen A. S. Berkovic S. F. Cossette P. Delanty N. (2013). De novo mutations in epileptic encephalopathies. *Nature* 501 217–221. 10.1038/nature12439 23934111 PMC3773011

[B24] Feldman D. E. (2012). The spike-timing dependence of plasticity. *Neuron* 75 556–571. 10.1016/j.neuron.2012.08.001 22920249 PMC3431193

[B25] Ferraro T. N. Golden G. T. Smith G. G. St Jean P. Schork N. J. Mulholland N. (1999). Mapping loci for pentylenetetrazol-induced seizure susceptibility in mice. *J. Neurosci.* 19 6733–6739. 10.1523/jneurosci.19-16-06733.1999 10436030 PMC6782858

[B26] Fromer M. Pocklington A. J. Kavanagh D. H. Williams H. J. Dwyer S. Gormley P. (2014). De novo mutations in schizophrenia implicate synaptic networks. *Nature* 506 179–184. 10.1038/nature12929 24463507 PMC4237002

[B27] Gazina E. V. Leaw B. T. Richards K. L. Wimmer V. C. Kim T. H. Aumann T. D. (2015). ‘Neonatal’ Nav1.2 reduces neuronal excitability and affects seizure susceptibility and behaviour. *Hum. Mol. Genet.* 24 1457–1468. 10.1093/hmg/ddu562 25378553

[B28] Gazina E. V. Richards K. L. Mokhtar M. B. Thomas E. A. Reid C. A. Petrou S. (2010). Differential expression of exon 5 splice variants of sodium channel alpha subunit mRNAs in the developing mouse brain. *Neuroscience* 166 195–200. 10.1016/j.neuroscience.2009.12.011 20006674

[B29] Geisheker M. R. Heymann G. Wang T. Coe B. P. Turner T. N. Stessman H. A. F. (2017). Hotspots of missense mutation identify neurodevelopmental disorder genes and functional domains. *Nat. Neurosci.* 20 1043–1051. 10.1038/nn.4589 28628100 PMC5539915

[B30] Golding N. L. Staff N. P. Spruston N. (2002). Dendritic spikes as a mechanism for cooperative long-term potentiation. *Nature* 418 326–331. 10.1038/nature00854 12124625

[B31] Gong B. Rhodes K. J. Bekele-Arcuri Z. Trimmer J. S. (1999). Type I and type II Na(+) channel alpha-subunit polypeptides exhibit distinct spatial and temporal patterning, and association with auxiliary subunits in rat brain. *J. Comp. Neurol.* 412 342–352. 10.1002/(sici)1096-9861(19990920)412:2<342::aid-cne11>3.0.co;2-2 10441760

[B32] Gustafson T. A. Clevinger E. C. O’Neill T. J. Yarowsky P. J. Krueger B. K. (1993). Mutually exclusive exon splicing of type III brain sodium channel alpha subunit RNA generates developmentally regulated isoforms in rat brain. *J. Biol. Chem.* 268 18648–18653. 8395514

[B33] Hamdan F. F. Myers C. T. Cossette P. Lemay P. Spiegelman D. Laporte A. D. (2017). High rate of recurrent de novo mutations in developmental and epileptic encephalopathies. *Am. J. Hum. Genet.* 101 664–685. 10.1016/j.ajhg.2017.09.008 29100083 PMC5673604

[B34] Heron S. E. Crossland K. M. Andermann E. Phillips H. A. Hall A. J. Bleasel A. (2002). Sodium-channel defects in benign familial neonatal-infantile seizures. *Lancet* 360 851–852. 10.1016/s0140-6736(02)09968-3 12243921

[B35] Howell K. B. McMahon J. M. Carvill G. L. Tambunan D. Mackay M. T. Rodriguez-Casero V. (2015). SCN2A encephalopathy: a major cause of epilepsy of infancy with migrating focal seizures. *Neurology* 85 958–966. 10.1212/WNL.0000000000001926 26291284 PMC4567464

[B36] Hu W. Tian C. Li T. Yang M. Hou H. Shu Y. (2009). Distinct contributions of Na(v)1.6 and Na(v)1.2 in action potential initiation and backpropagation. *Nat. Neurosci.* 12 996–1002. 10.1038/nn.2359 19633666

[B37] Iossifov I. O’Roak B. J. Sanders S. J. Ronemus M. Krumm N. Levy D. (2014). The contribution of de novo coding mutations to autism spectrum disorder. *Nature* 515 216–221. 10.1038/nature13908 25363768 PMC4313871

[B38] Jiang Y. H. Yuen R. K. Jin X. Wang M. Chen N. Wu X. (2013). Detection of clinically relevant genetic variants in autism spectrum disorder by whole-genome sequencing. *Am. J. Hum. Genet.* 93 249–263. 10.1016/j.ajhg.2013.06.012 23849776 PMC3738824

[B39] Johnson K. W. Herold K. F. Milner T. A. Hemmings H. C. Jr. Platholi J. (2017). Sodium channel subtypes are differentially localized to pre- and post-synaptic sites in rat hippocampus. *J. Comp. Neurol.* 525 3563–3578. 10.1002/cne.24291 28758202 PMC5927368

[B40] Johnston D. Christie B. R. Frick A. Gray R. Hoffman D. A. Schexnayder L. K. (2003). Active dendrites, potassium channels and synaptic plasticity. *Philos. Trans. R. Soc. Lond. B. Biol. Sci.* 358 667–674. 10.1098/rstb.2002.1248 12740112 PMC1693145

[B41] Jung H. Park H. Choi Y. Kang H. Lee E. Kweon H. (2018). Sexually dimorphic behavior, neuronal activity, and gene expression in Chd8-mutant mice. *Nat. Neurosci.* 21 1218–1228. 10.1038/s41593-018-0208-z 30104731

[B42] Kamiya K. Kaneda M. Sugawara T. Mazaki E. Okamura N. Montal M. (2004). A nonsense mutation of the sodium channel gene SCN2A in a patient with intractable epilepsy and mental decline. *J. Neurosci.* 24 2690–2698. 10.1523/jneurosci.3089-03.2004 15028761 PMC6729532

[B43] Kaplan M. R. Cho M. H. Ullian E. M. Isom L. L. Levinson S. R. Barres B. A. (2001). Differential control of clustering of the sodium channels Na(v)1.2 and Na(v)1.6 at developing CNS nodes of ranvier. *Neuron* 30 105–119. 10.1016/s0896-6273(01)00266-5 11343648

[B44] Kim R. Kim J. Chung C. Ha S. Lee S. Lee E. (2018). Cell-type-specific shank2 deletion in mice leads to differential synaptic and behavioral phenotypes. *J. Neurosci.* 38 4076–4092. 10.1523/JNEUROSCI.2684-17.2018 29572432 PMC6596028

[B45] Kim Y. Hsu C. L. Cembrowski M. S. Mensh B. D. Spruston N. (2015). Dendritic sodium spikes are required for long-term potentiation at distal synapses on hippocampal pyramidal neurons. *eLife* 4:e06414. 10.7554/eLife.06414 26247712 PMC4576155

[B46] Klassen T. Davis C. Goldman A. Burgess D. Chen T. Wheeler D. (2011). Exome sequencing of ion channel genes reveals complex profiles confounding personal risk assessment in epilepsy. *Cell* 145 1036–1048. 10.1016/j.cell.2011.05.025 21703448 PMC3131217

[B47] Koester H. J. Sakmann B. (1998). Calcium dynamics in single spines during coincident pre- and postsynaptic activity depend on relative timing of back-propagating action potentials and subthreshold excitatory postsynaptic potentials. *Proc. Natl. Acad. Sci. U.S.A.* 95 9596–9601. 10.1073/pnas.95.16.9596 9689126 PMC21384

[B48] Kole M. H. Ilschner S. U. Kampa B. M. Williams S. R. Ruben P. C. Stuart G. J. (2008). Action potential generation requires a high sodium channel density in the axon initial segment. *Nat. Neurosci.* 11 178–186. 10.1038/nn2040 18204443

[B49] Kole M. H. Stuart G. J. (2012). Signal processing in the axon initial segment. *Neuron* 73 235–247. 10.1016/j.neuron.2012.01.007 22284179

[B50] Krumm N. Turner T. N. Baker C. Vives L. Mohajeri K. Witherspoon K. (2015). Excess of rare, inherited truncating mutations in autism. *Nat. Genet.* 47 582–588. 10.1038/ng.3303 25961944 PMC4449286

[B51] Krupp D. R. Barnard R. A. Duffourd Y. Evans S. A. Mulqueen R. M. Bernier R. (2017). Exonic mosaic mutations contribute risk for autism spectrum disorder. *Am. J. Hum. Genet.* 101 369–390. 10.1016/j.ajhg.2017.07.016 28867142 PMC5590950

[B52] Larkum M. E. Zhu J. J. Sakmann B. (1999). A new cellular mechanism for coupling inputs arriving at different cortical layers. *Nature* 398 338–341. 10.1038/18686 10192334

[B53] Lee E. J. Lee H. Huang T. N. Chung C. Shin W. Kim K. (2015). Trans-synaptic zinc mobilization improves social interaction in two mouse models of autism through NMDAR activation. *Nat. Commun.* 6:7168. 10.1038/ncomms8168 25981743 PMC4479043

[B54] Li J. Wang L. Guo H. Shi L. Zhang K. Tang M. (2017). Targeted sequencing and functional analysis reveal brain-size-related genes and their networks in autism spectrum disorders. *Mol. Psychiatry* 22 1282–1290. 10.1038/mp.2017.140 28831199

[B55] Li T. Tian C. Scalmani P. Frassoni C. Mantegazza M. Wang Y. (2014). Action potential initiation in neocortical inhibitory interneurons. *PLoS Biol.* 12:e1001944. 10.1371/journal.pbio.1001944 25203314 PMC4159120

[B56] Liao Y. Deprez L. Maljevic S. Pitsch J. Claes L. Hristova D. (2010). Molecular correlates of age-dependent seizures in an inherited neonatal-infantile epilepsy. *Brain* 133 1403–1414. 10.1093/brain/awq057 20371507

[B57] Lussier M. P. Sanz-Clemente A. Roche K. W. (2015). Dynamic regulation of N-Methyl-d-aspartate (NMDA) and alpha-Amino-3-hydroxy-5-methyl-4-isoxazolepropionic acid (AMPA) receptors by posttranslational modifications. *J. Biol. Chem.* 290 28596–28603. 10.1074/jbc.R115.652750 26453298 PMC4661374

[B58] Magee J. C. Johnston D. (1997). A synaptically controlled, associative signal for hebbian plasticity in hippocampal neurons. *Science* 275 209–213. 10.1126/science.275.5297.209 8985013

[B59] Malenka R. C. Bear M. F. (2004). LTP and LTD: an embarrassment of riches. *Neuron* 44 5–21. 15450156 10.1016/j.neuron.2004.09.012

[B60] Middleton S. J. Kneller E. M. Chen S. Ogiwara I. Montal M. Yamakawa K. (2018). Altered hippocampal replay is associated with memory impairment in mice heterozygous for the Scn2a gene. *Nat. Neurosci.* 21 996–1003. 10.1038/s41593-018-0163-8 29867081 PMC7306226

[B61] Moore J. J. Ravassard P. M. Ho D. Acharya L. Kees A. L. Vuong C. (2017). Dynamics of cortical dendritic membrane potential and spikes in freely behaving rats. *Science* 355:eaaj1497. 10.1126/science.aaj1497 28280248

[B62] Nadler J. J. Moy S. S. Dold G. Trang D. Simmons N. Perez A. (2004). Automated apparatus for quantitation of social approach behaviors in mice. *Genes Brain Behav.* 3 303–314. 10.1111/j.1601-183x.2004.00071.x 15344923

[B63] Nakamura K. Kato M. Osaka H. Yamashita S. Nakagawa E. Haginoya K. (2013). Clinical spectrum of SCN2A mutations expanding to ohtahara syndrome. *Neurology* 81 992–998. 10.1212/WNL.0b013e3182a43e57 23935176

[B64] Naydenov A. V. Horne E. A. Cheah C. S. Swinney K. Hsu K. L. Cao J. K. (2014). ABHD6 blockade exerts antiepileptic activity in PTZ-induced seizures and in spontaneous seizures in R6/2 mice. *Neuron* 83 361–371. 10.1016/j.neuron.2014.06.030 25033180 PMC4136499

[B65] Nunes D. Kuner T. (2018). Axonal sodium channel NaV1.2 drives granule cell dendritic GABA release and rapid odor discrimination. *PLoS Biol.* 16:e2003816. 10.1371/journal.pbio.2003816 30125271 PMC6117082

[B66] Ogiwara I. Ito K. Sawaishi Y. Osaka H. Mazaki E. Inoue I. (2009). De novo mutations of voltage-gated sodium channel alphaII gene SCN2A in intractable epilepsies. *Neurology* 73 1046–1053. 10.1212/WNL.0b013e3181b9cebc 19786696 PMC2754324

[B67] Ogiwara I. Miyamoto H. Tatsukawa T. Yamagata T. Nakayama T. Atapour N. (2018). Nav1.2 haplodeficiency in excitatory neurons causes absence-like seizures in mice. *Commun. Biol.* 1:96. 10.1038/s42003-018-0099-2 30175250 PMC6115194

[B68] Parrini E. Marini C. Mei D. Galuppi A. Cellini E. Pucatti D. (2017). Diagnostic targeted resequencing in 349 patients with drug-resistant pediatric epilepsies identifies causative mutations in 30 different genes. *Hum. Mutat.* 38 216–225. 10.1002/humu.23149 27864847

[B69] Peitz M. Pfannkuche K. Rajewsky K. Edenhofer F. (2002). Ability of the hydrophobic FGF and basic TAT peptides to promote cellular uptake of recombinant Cre recombinase: a tool for efficient genetic engineering of mammalian genomes. *Proc. Natl. Acad. Sci. U.S.A.* 99 4489–4494. 10.1073/pnas.032068699 11904364 PMC123675

[B70] Planells-Cases R. Caprini M. Zhang J. Rockenstein E. M. Rivera R. R. Murre C. (2000). Neuronal death and perinatal lethality in voltage-gated sodium channel alpha(II)-deficient mice. *Biophys. J.* 78 2878–2891. 10.1016/s0006-3495(00)76829-9 10827969 PMC1300874

[B71] Quinn L. P. Stean T. O. Chapman H. Brown M. Vidgeon-Hart M. Upton N. (2006). Further validation of LABORAS using various dopaminergic manipulations in mice including MPTP-induced nigro-striatal degeneration. *J. Neurosci. Methods* 156 218–227. 10.1016/j.jneumeth.2006.03.013 16626808

[B72] Quinn L. P. Stean T. O. Trail B. Duxon M. S. Stratton S. C. Billinton A. (2003). LABORAS: initial pharmacological validation of a system allowing continuous monitoring of laboratory rodent behaviour. *J. Neurosci. Methods* 130 83–92. 10.1016/s0165-0270(03)00227-9 14583407

[B73] Rauch A. Wieczorek D. Graf E. Wieland T. Endele S. Schwarzmayr T. (2012). Range of genetic mutations associated with severe non-syndromic sporadic intellectual disability: an exome sequencing study. *Lancet* 380 1674–1682. 10.1016/S0140-6736(12)61480-9 23020937

[B74] Sanders S. J. Campbell A. J. Cottrell J. R. Moller R. S. Wagner F. F. Auldridge A. L. (2018). Progress in understanding and treating SCN2A-mediated disorders. *Trends Neurosci.* 41 442–456. 10.1016/j.tins.2018.03.011 29691040 PMC6015533

[B75] Sanders S. J. Murtha M. T. Gupta A. R. Murdoch J. D. Raubeson M. J. Willsey A. J. (2012). De novo mutations revealed by whole-exome sequencing are strongly associated with autism. *Nature* 485 237–241. 22495306 10.1038/nature10945 PMC3667984

[B76] Scattoni M. L. Crawley J. Ricceri L. (2009). Ultrasonic vocalizations: a tool for behavioural phenotyping of mouse models of neurodevelopmental disorders. *Neurosci. Biobehav. Rev.* 33 508–515. 10.1016/j.neubiorev.2008.08.003 18771687 PMC2688771

[B77] Shah B. S. Stevens E. B. Pinnock R. D. Dixon A. K. Lee K. (2001). Developmental expression of the novel voltage-gated sodium channel auxiliary subunit beta3, in rat CNS. *J. Physiol.* 534 763–776. 10.1111/j.1469-7793.2001.t01-1-00763.x 11483707 PMC2278751

[B78] Silverman J. L. Yang M. Lord C. Crawley J. N. (2010). Behavioural phenotyping assays for mouse models of autism. *Nat. Rev. Neurosci.* 11 490–502. 10.1038/nrn2851 20559336 PMC3087436

[B79] Spruston N. (2008). Pyramidal neurons: dendritic structure and synaptic integration. *Nat. Rev. Neurosci.* 9 206–221. 10.1038/nrn2286 18270515

[B80] Stessman H. A. Xiong B. Coe B. P. Wang T. Hoekzema K. Fenckova M. (2017). Targeted sequencing identifies 91 neurodevelopmental-disorder risk genes with autism and developmental-disability biases. *Nat. Genet.* 49 515–526. 10.1038/ng.3792 28191889 PMC5374041

[B81] Sugawara T. Tsurubuchi Y. Agarwala K. L. Ito M. Fukuma G. Mazaki-Miyazaki E. (2001). A missense mutation of the Na+ channel alpha II subunit gene Na(v)1.2 in a patient with febrile and afebrile seizures causes channel dysfunction. *Proc. Natl. Acad. Sci. U.S.A.* 98 6384–6389. 10.1073/pnas.111065098 11371648 PMC33477

[B82] Tammimies K. Marshall C. R. Walker S. Kaur G. Thiruvahindrapuram B. Lionel A. C. (2015). Molecular diagnostic yield of chromosomal microarray analysis and whole-exome sequencing in children with autism spectrum disorder. *JAMA* 314 895–903. 26325558 10.1001/jama.2015.10078

[B83] Tavassoli T. Kolevzon A. Wang A. T. Curchack-Lichtin J. Halpern D. Schwartz L. (2014). De novo SCN2A splice site mutation in a boy with autism spectrum disorder. *BMC Med. Genet.* 15:35. 10.1186/1471-2350-15-35 24650168 PMC3994485

[B84] Tian C. Wang K. Ke W. Guo H. Shu Y. (2014). Molecular identity of axonal sodium channels in human cortical pyramidal cells. *Front. Cell. Neurosci.* 8:297. 10.3389/fncel.2014.00297 25294986 PMC4172021

[B85] Touma M. Joshi M. Connolly M. C. Grant P. E. Hansen A. R. Khwaja O. (2013). Whole genome sequencing identifies SCN2A mutation in monozygotic twins with ohtahara syndrome and unique neuropathologic findings. *Epilepsia* 54 e81–e85. 10.1111/epi.12137 23550958 PMC3640694

[B86] Trimmer J. S. Rhodes K. J. (2004). Localization of voltage-gated ion channels in mammalian brain. *Annu. Rev. Physiol.* 66 477–519. 10.1146/annurev.physiol.66.032102.113328 14977411

[B87] Trujillano D. Bertoli-Avella A. M. Kandaswamy K. K. Weiss M. E. R. Köster J. Marais A. (2017). Clinical exome sequencing: results from 2819 samples reflecting 1000 families. *EJHG* 25 176–182. 10.1038/ejhg.2016.146 27848944 PMC5255946

[B88] Turner T. N. Hormozdiari F. Duyzend M. H. McClymont S. A. Hook P. W. Iossifov I. (2016). Genome sequencing of autism-affected families reveals disruption of putative noncoding regulatory DNA. *Am. J. Hum. Genet.* 98 58–74. 10.1016/j.ajhg.2015.11.023 26749308 PMC4716689

[B89] Vacher H. Mohapatra D. P. Trimmer J. S. (2008). Localization and targeting of voltage-dependent ion channels in mammalian central neurons. *Physiol. Rev.* 88 1407–1447. 10.1152/physrev.00002.2008 18923186 PMC2587220

[B90] Van Wart A. Matthews G. (2006). Impaired firing and cell-specific compensation in neurons lacking nav1.6 sodium channels. *J. Neurosci.* 26 7172–7180. 10.1523/jneurosci.1101-06.2006 16822974 PMC6673932

[B91] Wang F. Flanagan J. Su N. Wang L. C. Bui S. Nielson A. (2012). RNAscope: a novel in situ RNA analysis platform for formalin-fixed, paraffin-embedded tissues. *J. Mol. Diagn.* 14 22–29. 10.1016/j.jmoldx.2011.08.002 22166544 PMC3338343

[B92] Wang T. Guo H. Xiong B. Stessman H. A. F. Wu H. Coe B. P. (2016). De novo genic mutations among a Chinese autism spectrum disorder cohort. *Nat. Commun.* 7:13316. 10.1038/ncomms13316 27824329 PMC5105161

[B93] Wang X. Bey A. L. Katz B. M. Badea A. Kim N. David L. K. (2016). Altered mGluR5-homer scaffolds and corticostriatal connectivity in a Shank3 complete knockout model of autism. *Nat. Commun.* 7:11459. 10.1038/ncomms11459 27161151 PMC4866051

[B94] Warburton E. C. Brown M. W. (2015). Neural circuitry for rat recognition memory. *Behav. Brain Res.* 285 131–139. 10.1016/j.bbr.2014.09.050 25315129 PMC4383363

[B95] Weiss L. A. Escayg A. Kearney J. A. Trudeau M. MacDonald B. T. Mori M. (2003). Sodium channels SCN1A, SCN2A and SCN3A in familial autism. *Mol. Psychiatry* 8 186–194. 10.1038/sj.mp.4001241 12610651

[B96] Westenbroek R. E. Merrick D. K. Catterall W. A. (1989). Differential subcellular localization of the RI and RII Na+ channel subtypes in central neurons. *Neuron* 3 695–704. 10.1016/0896-6273(89)90238-9 2561976

[B97] Wohr M. (2014). Ultrasonic vocalizations in shank mouse models for autism spectrum disorders: detailed spectrographic analyses and developmental profiles. *Neurosci. Biobehav. Rev.* 43C, 199–212. 10.1016/j.neubiorev.2014.03.021 24726578

[B98] Wolff M. Johannesen K. M. Hedrich U. B. S. Masnada S. Rubboli G. Gardella E. (2017). Genetic and phenotypic heterogeneity suggest therapeutic implications in SCN2A-related disorders. *Brain* 140 1316–1336. 10.1093/brain/awx054 28379373

[B99] Won H. Lee H. R. Gee H. Y. Mah W. Kim J. I. Lee J. (2012). Autistic-like social behaviour in shank2-mutant mice improved by restoring NMDA receptor function. *Nature* 486 261–265. 10.1038/nature11208 22699620

[B100] Yamagata T. Ogiwara I. Mazaki E. Yanagawa Y. Yamakawa K. (2017). Nav1.2 is expressed in caudal ganglionic eminence-derived disinhibitory interneurons: mutually exclusive distributions of Nav1.1 and Nav1.2. *Biochem. Biophys. Res. Commun.* 491 1070–1076. 10.1016/j.bbrc.2017.08.013 28784306

[B101] Yokoi T. Enomoto Y. Tsurusaki Y. Naruto T. Kurosawa K. (2018). Nonsyndromic intellectual disability with novel heterozygous SCN2A mutation and epilepsy. *Hum. Genome Var.* 5:20. 10.1038/s41439-018-0019-5 30062040 PMC6054605

[B102] Yoo T. Cho H. Lee J. Park H. Yoo Y. E. Yang E. (2018). GABA neuronal deletion of shank3 exons 14-16 in mice suppresses striatal excitatory synaptic input and induces social and locomotor abnormalities. *Front. Cell. Neurosci.* 12:341. 10.3389/fncel.2018.00341 30356810 PMC6189516

[B103] Yuen R. K. Thiruvahindrapuram B. Merico D. Walker S. Tammimies K. Hoang N. (2015). Whole-genome sequencing of quartet families with autism spectrum disorder. *Nat. Med.* 21 185–191. 10.1038/nm.3792 25621899

